# Analysis of optimal control strategies on the fungal *Tinea capitis* infection fractional order model with cost-effective analysis

**DOI:** 10.1038/s41598-024-51670-y

**Published:** 2024-01-17

**Authors:** Shewafera Wondimagegnhu Teklu, Abebe Addise Meshesha, Saif Ullah

**Affiliations:** 1 Department of Mathematics, College of Natural and Computational Sciences, Debre Berhan University, 445 Debre Berhan, Ethiopia; 2 Department of Surgery, College of Health Science, Debre Berhan University, 445 Debre Berhan, Ethiopia; 3 Department of Mathematics, University of Peshawar, Peshawar, Khyber Pakhtunkhwa Pakistan

**Keywords:** Computational biology and bioinformatics, Medical research, Pathogenesis, Mathematics and computing

## Abstract

In this study, we have formulated and analyzed the *Tinea capitis* infection Caputo fractional order model by implementing three time-dependent control measures. In the qualitative analysis part, we investigated the following: by using the well-known Picard–Lindelöf criteria we have proved the model solutions' existence and uniqueness, using the next generation matrix approach we calculated the model basic reproduction number, we computed the model equilibrium points and investigated their stabilities, using the three time-dependent control variables (prevention measure, non-inflammatory infection treatment measure, and inflammatory infection treatment measure) and from the formulated fractional order model we re-formulated the fractional order optimal control problem. The necessary optimality conditions for the *Tinea capitis* fractional order optimal control problem and the existence of optimal control strategies are derived and presented by using Pontryagin’s Maximum Principle. Also, the study carried out the sensitivity and numerical analysis to investigate the most sensitive parameters and to verify the qualitative analysis results. Finally, we performed the cost-effective analysis to investigate the most cost-effective measures from the possible proposed control measures, and from the findings we can suggest that implementing prevention measures only is the most cost-effective control measure that stakeholders should consider.

## Introduction

Anthropophilic (human), zoophilic (animal), or geophilic (soil) are Dermatophytes classifications with their species as Microsporum,Trichophyton, and Epidermophyton and also they are the main causes of Dermatophytosis^1,2^. Dermatophyte is one of the most common causes of human fungal infections throughout the world and affecting the skin, hair, scalp, and nails of human and animal hosts^1,3^.

*Tinea capitis* (*T. capitis*) is one of the most common contagious dermatophyte superficial fungal infection of the scalp hairs and intervening skin of human beings but commonly observed in people of African descent as compared to Caucasians and Hispanics and it has been considered a significant public health issue for decades and appears most often in children between 3 and 7 years of age^3–9^. *Tinea capitis* can be classified as an inflammatory or non-inflammatory fungal dermatophyte infection caused by fungal species like Trichophyton or Microsporum or zoophilic or anthropophilic and affecting the hair and scalp skin of mainly among prepubertal children^10–21^. Non-inflammatory *Tinea capitis* infection is not usually causes a permanent hair loss but cause a black dot tinea captis where the individual’s hair shafts break at the scalp area, whereas inflammatory *Tinea capitis* infection is a manifestation of acquired immunity and it can be classified as: Majocchi’s granuloma, kerion Celsi and favus^2–6^.

It is often ignored but it accounts for 25–30% of all fungal infections and is common in developing countries especially in sub-Saharan Africa countries^13,14^. The *Tinea capitis* is a common children hair or scalp infection problem, and has been a worldwide public health concern and since it is a highly contagious infection and it has adverse events such as high economic burden, blood test follow-up, and significant cost of systemic treatments^1,15^. It predominantly occurs in rular or suburban regions and its common risk factors are poor hygiene, overcrowding, humidity, cultural habits, socioeconomic status and heat^2,15,20^. Even though, it is not always possible to carry out the *Tinea capitis* infection test, the etiological diagnosis is depending on the patients’ clinical findings and confirmation based on the fungus growth in culture^17^. The dermatoscopic and clinical findings of the tinea suspected cases help to identify the specific treatment, facilitating precocious and the etiological agent^17^. Antifungal oral therapy (griseofulvin, terbinafine, fluconazole, and itraconazole) has been considered the basic standard for the fungal *Tinea capitis* infection^2^.

Analyses of mathematical modeling for real world situations have been the most important tool in understanding of different aspects of the real world phenomenon^22,23^. Different researchers have been formulated and analyzed a mathematical model for system dynamics in different research topics such as social sciences, natural sciences and other sciences, see scholars studies^24–54^. We have found various literatures done by different researchers that have investigated the real world situations phenomenon using integer order modeling approach like^22–24,37–40^, using fractional order modeling approach like^25–27,33–36,46–48^.

From our reviewing literature process we have faced difficulty on finding published research about fungal *Tinea capitis* infection spreading with mathematical modeling approach, getting organized real data about *Tinea capitis* infection. But we have found different published literatures with cross-sectional approach, case report approach and notes prepared by various organizations that are used to justify the current and previous incidence and prevalence conditions and also to observe its spreading nature. Having these in mind we have reviewed the following studies about different real world situations that are more relevant and some of them are related to the proposed *Tinea capitis* disease study regarding to basic concepts such as mathematical modelling approach, theories, methods and methodologies. Karanja et al.^16^, constructed analyzed a ringworm infections deterministic mathematical model in an environment. The analysis shows that the ringworm dynamical system is globally asymptotically stable when the basic reproduction number is less than one. Alemneh et al.^24^ formulated and analyzed an integer order model on social media addiction to investigate the most effective strategies to tackle the problem. Teklu and Terefe^38^ and Teklu^39^ formulated and analyzed an integer order model and a fractional order model to investigate the most effective strategies on the transmission dynamics of university students’ animosity towards mathematics and anxiety towards mathematics respectively. Results of both the studies show that using protection and treatment control strategies simultaneously is the best strategy to minimize the transmission dynamics of either animosity or anxiety of mathematics in the community. Mandal et al.^34^ constructed and examined a fractional-order epidemic model with fear effect of a communicable disease with treatment control measure. The study analyzed fractional backward and fractional Hopf bifurcation and determined possible roles of the disease control parameters, level of fear. Din et al.^25^ formulated and examined a Caputo fractional order model on climate change. The study analyzed the model both qualitatively and numerically and the results show that the total spectrum lying between two integer values are achieved with more information about the complexity of the fractional climate change-model dynamics. Kotola and Teklu^32^ developed and analyzed a racism and corruption co-dynamics as infectious diseases and investigate the role of controlling mechanisms. Teklu and Terefe^22^ constructed and analyzed a violence and racism co-dynamics as contagious diseases and investigate the role of controlling mechanisms. From the analysis results one can observed that the violence-racism co-existence spreads under control if the co-existence corresponding basic reproduction number is less than one, and it propagates through the community if this number exceeds unity. Teklu et al.^54^ developed and investigated *Tinea capitis* epidemic fractional order model with optimal control theory. Their model did not consider non-inflammatory and inflammatory groups and cost-effective analysis. Mamo^49^ formulated and analyzed the transmission dynamics of racism in cyberspace. The analysis results show that the racism spreading could be under control whenever the corresponding basic reproduction number is less than unity and it spreads in the community whenever the basic reproduction number is more than unity. Similarly Mamo^44^ formulated and examined an integer order deterministic model on the transmission dynamics of racism propagation with community resilience. One can observed the result when decreasing the transmission and racial extremeness rate by increasing the social bonds and solidarity through society resilience could control the transmission dynamics of racism in the community. From the findings of the above studies one can observe that the fractional order derivative method could produce better solutions in the comparison the classical (integer order order) counterpart models, but the analysis of fractional order method is more complicated than the classical integer order approach.

Since we have understand that fungal *Tinea capitis* infection is the most common infectious disease attacking mainly children from 3 to 7 years old we are motivated to investigates fungal *Tinea capitis* infection spreading dynamics using fractional order optimal control problem by considering three time-dependent control measures. In the model formulation, the fractional order derivative approach in the Caputo case is considered. Moreover, the human population of infected group is partitioned into non-inflammatory infected and inflammatory infected sub-groups. The control measures we considered are prevention against *Tinea capitis* infection, treatment of non-inflammatory infected individuals and treatment of inflammatory infected individuals. We performed numerical simulations for the fractional order optimal control problem in order to investigate the impact of the control measures. The main aim of this study is formulating and examining the Caputo fractional order model of the *Tinea capitis* spreading dynamics with optimal control theory.

According to various mathematical modeling research studies of infectious diseases discussed above, none of them considered a fractional order mathematical model study on *Tinea capitis* with non-inflammatory and inflammatory infection classification to tackle the spreading dynamics in the community by applying the prevention and treatment optimal control strategies with minimum cost. As a result, this scientific gap motivates us to formulate and investigate a novel fractional order mathematical model of on fungal *Tinea capitis* infection spreading in the community. The rest part of this paper is organized in different sections as: “Basic concepts of fractional calculus” section addressed the fundamental concepts regarding fractional order calculus, “Qualitative analysis of the fractional order model (16)” section discussed procedures of the models formulations and analyzed the *Tinea capitis* transmission dynamics Caputo fractional-order model, “Formulation of the corresponding optimal control problem” section re-formulated and analyzed the Caputo fractional order optimal control problem, “Sensitivity and numerical analysis” section carried out the sensitivity and numerical analysis of the fractional order model, “Cost-effective analysis” section investigate the cost-electiveness analysis, and finally “Discussion and conclusion” section gives the conclusion of the whole activities in the study.

## Basic concepts of fractional calculus

In mathematical modeling of real world situations especially in mathematical epidemiology the concept of fractional calculus is a fundamental tool. Having this in mind, in this section we illustrate the basic aspects of fractional calculus (both the integral and derivative aspects) that are relevant to our *Tinea capitis* infection investigation with subsequent sections and sub-sections.

### Definition 1

Suppose $g\left( t \right) \in L^{\infty } \left( {\mathbb{R}} \right) \cap {\mathcal{F}}\left( {\mathbb{R}} \right).$ Let $\kappa > 0$ then the Riemann–Liouville fractional integral of order $\kappa > 0$ is defined by

1 $$ I_{a}^{\kappa } f\left( t \right) = \frac{1}{{\Gamma \left( {\upkappa } \right)}}\mathop \smallint \limits_{a}^{t} \left( {t - \tau } \right)^{\kappa - 1} g\left( \tau \right)d\tau ,\;t > 0, $$

where $\Gamma \left( . \right)$ is the gamma function^25^.

### Definition 2

Suppose $g\left( t \right) \in L^{\infty } \left( {\mathbb{R}} \right) \cap {\mathcal{F}}\left( {\mathbb{R}} \right)$ then the Riemann–Liouville type fractional order derivative of $g$ having order $\kappa > 0$ is defined by

2 $$^{{{\text{RL}}}} D_{t}^{\kappa } g\left( t \right) = \left\{ {\begin{array}{*{20}l} {\frac{1}{{\Gamma \left( {{\text{m}} - {\upkappa }} \right)}}\frac{{d^{m} }}{{dt^{m} }}\mathop \smallint \limits_{0}^{t} \left( {t - \tau } \right)^{m - \kappa - 1} g\left( \tau \right)d\tau ,} \hfill & {m - 1 < \kappa \le m \in N} \hfill \\ {\frac{{d^{m} }}{{dt^{m} }}g\left( t \right),} \hfill & {\kappa = m \in N} \hfill \\ \end{array} } \right.,\;t > 0, $$

where ${\Gamma }\left( . \right)$ is the gamma function^53^.

### Definition 3

Suppose $g\left( t \right) \in L^{\infty } \left( {\mathbb{R}} \right) \cap {\mathcal{F}}\left( {\mathbb{R}} \right)$ then the Caputo type fractional order derivative of $g$ having order $\kappa > 0$ is defined by

3 $$^{{\text{C}}} D_{t}^{\kappa } g\left( t \right) = \left\{ {\begin{array}{*{20}l} {\frac{1}{{\Gamma \left( {{\text{m}} - {\upkappa }} \right)}}\mathop \smallint \limits_{0}^{t} \left( {t - \tau } \right)^{m - \kappa - 1} g^{m} \left( \tau \right)d\tau ,} \hfill & {m - 1 < \kappa \le m \in N} \hfill \\ {\frac{{d^{m} }}{{dt^{m} }}g\left( t \right),} \hfill & {\kappa = m \in N} \hfill \\ \end{array} } \right.,\;t > 0, $$

where $\Gamma \left( . \right)$ is the gamma function^53^.

### Proposition l

Stated below summaries some fundamental representations on the Riemann–Liouville integral illustrated in (1), the Riemann–Liouville and Caputo fractional operator illustrated in Eqs. (2) and (3) respectively.

### Proposition 2

Suppose $g\left( t \right) \in L^{\infty } \left( {\mathbb{R}} \right) \cap {\mathcal{F}}\left( {\mathbb{R}} \right)$ and $\kappa \in {\mathbb{R}},$ $m - 1 < \kappa \le m, m \in N$ the the following conditions hold

- $$\left( {^{{\text{C}}} D_{t}^{\kappa } I^{\kappa } g} \right)\left( t \right) = g\left( t \right).$$
- $$(I^{\kappa } \;^{{\text{C}}} D_{t}^{\kappa } g)\left( t \right) = g\left( t \right) - \mathop \sum \limits_{k = 0}^{m - 1} \frac{{t^{i} }}{i!}g^{i} \left( 0 \right).$$
- Especially, if $0 < \kappa < 1$, then

4 $$ (I^{\kappa } \;^{{\text{C}}} D_{t}^{\kappa } g)\left( t \right) = g\left( t \right) - g\left( 0 \right). $$

(d) For a constant function $g\left( t \right) = b$ then $^{{\text{C}}} D_{t}^{\kappa } \left( b \right) = 0$, where $m = \left[ \kappa \right] + 1$, with $\left[ \kappa \right]$ is the integer part of $\kappa \in {\mathbb{R}}_{ + }$ ^53^.

### Definition 4

Let $\alpha > 0,\beta > 0$ then the two parameters Mittag–Leffler function is defined by^47^

5 $$ E_{\alpha ,\beta } \left( t \right) = \mathop \sum \limits_{m = 0}^{\infty } \frac{{t^{m} }}{{{\Gamma }\left( {\alpha {\text{m}} + \beta } \right)}}. $$

### Definition 5

Suppose $\beta = 1$ be the constant parameter then the one parameter Mittag–Leffler function is defined by ^47^

6 $$ E_{\alpha ,1} \left( t \right) = \mathop \sum \limits_{m = 0}^{\infty } \frac{{t^{m} }}{{{\Gamma }\left( {\alpha {\text{m}} + 1} \right)}} = E_{\alpha } \left( t \right). $$

### Definition 6

A constant number $x^{*}$ is said to be an equilibrium point of the Caputo-fractional order model, then ^53^

7 $$^{{\text{C}}} D_{t}^{\kappa } x\left( t \right) = f\left( {t,x\left( t \right)} \right),\;\kappa \in \left[ {0,1} \right]\;{\text{if}}\;{\text{and}}\;{\text{only}}\;{\text{if}}\;f\left( {t,x^{*} } \right) = 0 $$

### Proposition 3

The Laplace transform of the Caputo fractional order derivative with order $\kappa ,$ $m - 1 < \kappa \le m, m \in N$ is given by

8 $$ L\left( {D_{t}^{\kappa } h} \right)\left( s \right) = s^{\kappa } H\left( s \right) - \mathop \sum \limits_{i = 1}^{m - 1} s^{\kappa - i - 1} h^{i} \left( 0 \right) $$

where $H\left( s \right)$ is the Laplace transform of the function $h\left( t \right)$ ^47^

### Proposition 4

The Laplace transformation of two parameters function of Mittag Leffler case is given by ^47^

9 $$ {\text{L}}\left( {{\text{t}}^{{\upbeta - 1}} {\text{ E}}_{{\upalpha ,\upbeta }} \left( { \pm\upgamma {\text{t}}^{\upalpha } } \right)} \right)\left( {\text{s}} \right) = \frac{{{\text{s}}^{{\upalpha \upbeta }} }}{{{\text{s}}^{\upalpha } \mp\upgamma }}. $$

### Proposition 5

(Generalized Mean Value theorem) Suppose $h\left( t \right) \in {\mathcal{L}}\left[ {0,T_{0} } \right]$ and ^C^ $D_{t}^{\kappa } h\left( t \right) \in {\mathcal{L}}\left[ {0,T_{0} } \right]$ for $\kappa \in \left( {0,1} \right]$ then the theorem states that

10 $$ h\left( t \right) = h\left( 0 \right) + \frac{1}{{{\Gamma }\left( \kappa \right)}}\;^{{\text{C}}} D_{t}^{\kappa } h\left( \xi \right)t^{\kappa } $$

where $\xi \in \left[ {0,t} \right]$, for each $t$ such that $0 < t \le T_{0}$ ^36^.

### Lemma 1

From proposition 3 we derived the following ^53^

11 $$ \begin{array}{*{20}l} {({\text{a}})} \hfill & {{\text{The}}\;{\text{function}}\;h\;{\text{is}}\;{\text{non - decreasing}}\;{\text{for}}\;{\text{all}}\;t \in \left[ {0,T_{0} } \right],\;{\text{if}}\;^{{\text{C}}} D_{t}^{\kappa } h\left( t \right) \ge 0.} \hfill \\ {({\text{b}})} \hfill & {{\text{The}}\;{\text{function}}\;h\;{\text{is}}\;{\text{non - decreasing}}\;{\text{for}}\;{\text{all}}\;t \in \left[ {0,T_{0} } \right],\;{\text{if}}\;^{{\text{C}}} D_{t}^{\kappa } h\left( t \right) \le 0.} \hfill \\ \end{array} $$

## Construction of the dynamical system

In this sub-section, we need to formulate both the integer order and fractional order representation of the fungal *Tinea capitis* infection spreading dynamics in the community by partitioning the human host population $K\left( t \right)$ into five distinct mutually exclusive groups as: $S\left( t \right)$ is the *Tinea capitis* infection susceptible group,$E\left( t \right)$ is the *Tinea capitis* exposed group, $U\left( t \right)$ is *Tinea capitis* non-inflammatory infected group, $A\left( t \right)$ is the *Tinea capitis* inflammatory group and $R\left( t \right)$ be the *Tinea capitis* infection recovered group where the total host population is given by

12 $$ K\left( t \right) = S\left( t \right) + E \left( t \right) + U \left( t \right) + A\left( t \right) + R\left( t \right). $$

Since the host population is not constant, the assumed host population is large and *Tinea capitis* is not a density dependent spreading infectious disease the *Tinea capitis* susceptible group acquire *Tinea capitis* infection at the standard incidence rate stated by

13 $$\uplambda _{{\text{T}}} \left( {\text{t}} \right) = \frac{{{\upvarphi }}}{K}\left( {\uprho _{1} U\left( t \right) +\uprho _{2} A\left( t \right)} \right), $$

where $\uprho _{1}$ and $\uprho _{2}$ are relative infectiousness of non-inflammatory and inflammatory infected groups respectively.

Additional fundamental model assumptions:

- A portion $\pi$ of the exposed group go to the *Tinea capitis* inflammatory group $A\left( t \right)$ at rate $\delta$.
- The remaining portion of exposed group given by $1 - \pi$ of the rate $\delta$ go to the non-inflammatory group $U\left( t \right).$
- Some of the individuals from the non-inflammatory group $U\left( t \right)$ and the inflammatory group $A\left( t \right)$ are entering to the *Tinea capitis* recovered group $R\left( t \right)$ with rates $\varepsilon_{1}$ and $\varepsilon_{2}$ respectively.
- The total host population is not constant.
- There is permanent recovery from *Tinea capitis*.
- The host population is homogeneously mixing.

**Figure 1:**
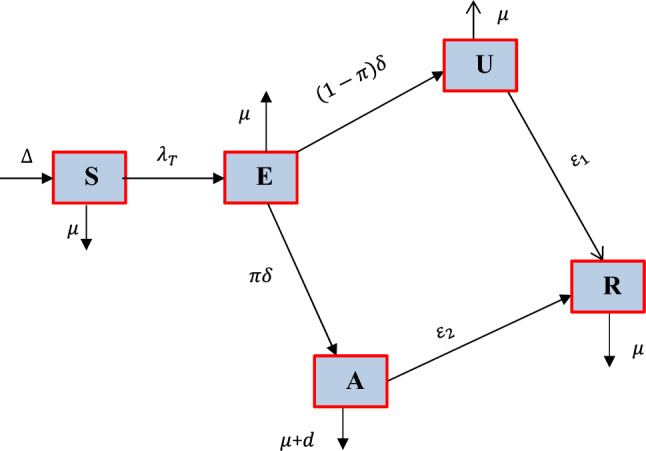
Schematic diagram of the *Tinea capitis* transmission where $\lambda_{T}$ stated in Eq. (13).

Applying each basic terminology illustrated above and descriptions in Tables 1 and 2 respectively the schematic diagram representation of the *Tinea capitis* transmission dynamics is given by Fig. 1.

**Table 1:** Definitions of the model parameters.

Symbol	Definition
$\mu$	The human host natural death rate
${\Delta }$	Host recruitment rate
$\delta$	Rate of the exposed group progress to the infectious groups
$\varepsilon_{1}$	Recovery rate of non-inflammatory group
$\varepsilon_{2}$	Recovery rate of inflammatory group
$\phi$	*Tinea capitis* spreading rate
$\pi$	Portion of exposed who are entered to inflammatory group
$d$	Inflammatory infection induced death rate

**Table 2:** State variables definitions.

Symbol	Definition
$S$	Susceptible to *Tinea capitis* group
$E$	*Tinea capitis* exposed group
$U$	*Tinea capitis* non-inflammatory infection group
$A$	Inflammatory *Tinea capitis* infection group
$R$	Recovered group against *Tinea capitis* infection

Applying the schematic diagram illustrated by Fig. 1 we have formulated the integer order model for *Tinea capitis* infection spreading in the community by the systems of non-linear ordinary differential equation given by

14 $$ \begin{gathered} \frac{dS}{{dt}} = {\Delta } - \left( {\lambda_{T} + \mu } \right)S, \hfill \\ \frac{dE}{{dt}} = \lambda_{T} {\text{S}} - \left( {\mu + \delta } \right)E, \hfill \\ \frac{{{\text{d}}U}}{{{\text{dt}}}} = \left( {1 -\uppi } \right)\updelta {\text{E}} - \left( {\upmu +\upvarepsilon _{1} } \right)U, \hfill \\ \frac{{{\text{d}}A}}{{{\text{dt}}}} = \uppi \updelta {\text{E}} - \left( {{\upmu } + d +\upvarepsilon _{2} } \right)A, \hfill \\ \frac{dR}{{dt}} =\upvarepsilon _{1} U +\upvarepsilon _{2} A - \mu R, \hfill \\ \end{gathered} $$

with initial host population

15 $$ S\left( 0 \right) > 0,\;E\left( 0 \right) \ge 0, U\left( 0 \right) \ge 0,\;A\left( 0 \right) \ge 0,\;{\text{and}}\; R\left( 0 \right) \ge 0. $$

Using the integer order *Tinea capitis* infection model (14) we re-construct the associated *Tinea capitis* spreading dynamics Caputo fractional order model in order to investigate the memory effects and gain more insights about the *Tinea capitis* spreading dynamics in the population. The *Tinea capitis* fractional order model is re-formulated as

16 $$ \begin{aligned} &^{{\text{C}}} D_{t}^{\kappa } S = {\Delta }^{\kappa } - \left( {{ }\lambda_{T} + \mu^{\kappa } } \right)S, \\ &^{{\text{C}}} D_{t}^{\kappa } E = \lambda_{T} {\text{S}} - \left( {\mu^{\kappa } + \delta^{\kappa } } \right)E, \\ &^{{\text{C}}} D_{t}^{\kappa } U = \pi^{\kappa } \delta^{\kappa } E - \left( {\mu^{\kappa } + \varepsilon_{1}^{\kappa } } \right), \\ &^{{\text{C}}} D_{t}^{\kappa } A = \left( {1 - \pi^{\kappa } } \right)\delta^{\kappa } E - \left( {\upmu ^{\kappa } + d^{\kappa } + \varepsilon_{2}^{\kappa } } \right)A, \\ &^{{\text{C}}} D_{t}^{\kappa } R = \varepsilon_{1}^{\kappa } U + \varepsilon_{2}^{\kappa } A - \mu^{\kappa } R \\ \end{aligned} $$

with initial host population described by

17 $$ \begin{aligned} & S\left( 0 \right) \ge 0,\;E\left( 0 \right) \ge 0,\;U\left( 0 \right) \ge 0, \\ & A\left( 0 \right) \ge 0,\;R\left( 0 \right) \ge 0. \\ \end{aligned} $$

## Qualitative analysis of the fractional order model (16)

### The model (16) solutions existence and uniqueness

Let $T_{F}$ be a positive real number and $J = \left[ {0,T_{F} } \right]$. Let ${\mathcal{F}}_{b}^{0} \left( J \right)$ is the set of every bounded continuous function defined on $J$ with the associtated norm defined by $W = sup\left\{ {\left| {W\left( t \right)} \right|:t \in J} \right\}.$ The dynamical system with Eq. (12) can be defined in the form

18 $$ \left\{ {\begin{array}{*{20}l} {D_{t}^{\kappa } W\left( t \right) = E\left( {t,W\left( t \right)} \right), 0 < t < T_{F} < \infty ,} \hfill \\ {W\left( 0 \right) = W_{0} ,} \hfill \\ \end{array} } \right. $$

in which $W\left( t \right) = \left( {S\left( t \right),E\left( t \right),U\left( t \right),A\left( t \right),R\left( t \right)} \right)$ describes the dynamical system (16) host population sub-divisions and $E$ is a continuous function given by

19 $$ E\left( {t,W\left( t \right)} \right) = \left[ {\begin{array}{*{20}c} {E_{1} \left( {t,S\left( t \right)} \right)} \\ {E_{2} \left( {t,E\left( t \right)} \right)} \\ {E_{3} \left( {t,U\left( t \right)} \right)} \\ {E_{4} \left( {t,A\left( t \right)} \right)} \\ {E_{5} \left( {t,R\left( t \right)} \right)} \\ \end{array} } \right] = \left[ {\begin{array}{*{20}c} {{\Delta }^{\kappa } - \left( {{ }\lambda_{T} + \mu^{\kappa } } \right)S} \\ {\lambda_{T} S - \left( {\mu^{\kappa } + \delta^{\kappa } } \right)E} \\ {(1 - \pi^{\kappa } )\delta^{\kappa } E - \left( {\mu^{\kappa } + \varepsilon_{1}^{\kappa } } \right)U} \\ {\pi^{\kappa } \delta^{\kappa } E - \left( {{\upmu }^{\kappa } + d^{\kappa } + \varepsilon_{2}^{\kappa } } \right)A} \\ {\varepsilon_{1}^{\kappa } U + \varepsilon_{2}^{\kappa } A - \mu^{\kappa } R} \\ \end{array} } \right]. $$

Using term (c) of Proposition 2 we have derived the following integral equations

20 $$ \begin{aligned} & S\left( t \right) - S\left( 0 \right) = I_{t}^{\kappa } \left( {{\Delta }^{\kappa } - \left( {{ }\lambda_{T} + \mu^{\kappa } } \right)S} \right), \\ & E\left( t \right) - E\left( 0 \right) = I_{t}^{\kappa } \left( {\lambda_{T} {\text{S}} - \left( {\mu^{\kappa } + \delta^{\kappa } } \right)E} \right), \\ & U\left( t \right) - U\left( 0 \right) = I_{t}^{\kappa } \left( {(1 - \pi^{\kappa } } \right)\delta^{\kappa } E - \left( {\mu^{\kappa } + \varepsilon_{1}^{\kappa } } \right)U), \\ & A\left( t \right) - A\left( 0 \right) = I_{t}^{\kappa } \left( {\pi^{\kappa } \delta^{\kappa } E - \left( {{\upmu }^{\kappa } + d^{\kappa } + \varepsilon_{2}^{\kappa } } \right)A} \right), \\ & R\left( t \right) - R\left( 0 \right) = I_{t}^{\kappa } \left( {\varepsilon_{1}^{\kappa } U + \varepsilon_{2}^{\kappa } A - \mu^{\kappa } R} \right). \\ \end{aligned} $$

From the system (15) we have derived the new system given by

21 $$ \begin{aligned} & S\left( t \right) = S\left( 0 \right) + \frac{1}{{{\Gamma }\left( \kappa \right)}}\mathop \smallint \limits_{0}^{t} \left( {t - s} \right)^{\kappa - 1} E_{1} \left( {s,S\left( s \right)} \right)ds, \\ & E\left( t \right) = E\left( 0 \right) + \frac{1}{{{\Gamma }\left( \kappa \right)}}\mathop \smallint \limits_{0}^{t} \left( {t - s} \right)^{\kappa - 1} E_{2} \left( {s,E\left( s \right)} \right)ds, \\ & U\left( t \right) = U\left( 0 \right) + \frac{1}{{{\Gamma }\left( \kappa \right)}}\mathop \smallint \limits_{0}^{t} \left( {t - s} \right)^{\kappa - 1} E_{3} \left( {s,U\left( s \right)} \right)ds, \\ & A\left( t \right) = A\left( 0 \right) + \frac{1}{{{\Gamma }\left( \kappa \right)}}\mathop \smallint \limits_{0}^{t} \left( {t - s} \right)^{\kappa - 1} E_{4} \left( {s,A\left( s \right)} \right)ds, \\ & R\left( t \right) = R\left( 0 \right) + \frac{1}{{{\Gamma }\left( \kappa \right)}}\mathop \smallint \limits_{0}^{t} \left( {t - s} \right)^{\kappa - 1} E_{5} \left( {s,R\left( s \right)} \right)ds. \\ \end{aligned} $$

Furthermore, by applying the Picard’s numerical iteration criteria we have described the iterated integral equations given by

22 $$ \begin{aligned} & S_{n} \left( t \right) = \frac{1}{{{\Gamma }\left( \kappa \right)}}\mathop \smallint \limits_{0}^{t} \left( {t - s} \right)^{\kappa - 1} E_{1} \left( {s,S_{n - 1} \left( s \right)} \right)ds, \\ & E_{n} \left( t \right) = \frac{1}{{{\Gamma }\left( \kappa \right)}}\mathop \smallint \limits_{0}^{t} \left( {t - s} \right)^{\kappa - 1} E_{2} \left( {s,E_{n - 1} \left( s \right)} \right)ds, \\ & I_{An} \left( t \right) = \frac{1}{{{\Gamma }\left( \kappa \right)}}\mathop \smallint \limits_{0}^{t} \left( {t - s} \right)^{\kappa - 1} E_{3} \left( {s,U_{n - 1} \left( s \right)} \right)ds, \\ & I_{Cn} \left( t \right) = \frac{1}{{{\Gamma }\left( \kappa \right)}}\mathop \smallint \limits_{0}^{t} \left( {t - s} \right)^{\kappa - 1} E_{4} \left( {s,A_{n - 1} \left( s \right)} \right)ds, \\ & R_{n} \left( t \right) = \frac{1}{{{\Gamma }\left( \kappa \right)}}\mathop \smallint \limits_{0}^{t} \left( {t - s} \right)^{\kappa - 1} E_{5} \left( {s,R_{n - 1} \left( s \right)} \right)ds. \\ \end{aligned} $$

Thus, the initial value problem (13) can be written by $W\left( t \right) = W\left( 0 \right) + \frac{1}{{{\Gamma }\left( \kappa \right)}}\mathop \smallint \limits_{0}^{t} E\left( {s, W\left( s \right)} \right)\left( {t - s} \right)^{\kappa - 1} ds$ and hence one can write the following Lemmas.

#### Lemma 2

The vector ${\text{E}}\left( {{\text{t}},{\text{W}}} \right)$ stated by Eq. (14) holds the Lipchitz condition in the variable ${\text{W}}$ on a set $\left[ {0,{\text{T}}_{{\text{F}}} } \right] \times {\mathbb{R}}_{ + }^{5}$ with Lipchitz constant $\Gamma = \max \left( {{{\upvarphi }}^{\kappa } \left( {\uprho _{1}^{{\upkappa }} +\uprho _{2}^{{\upkappa }} } \right),\;\left( {\upmu ^{{\upkappa }} +\updelta ^{{\upkappa }} } \right),\left( {\upmu ^{{\upkappa }} +\upvarepsilon _{1}^{{\upkappa }} } \right),\left( {\upmu ^{{\upkappa }} + {\text{d}}^{{\upkappa }} +\upvarepsilon _{2}^{{\upkappa }} } \right),\upmu ^{{\upkappa }} } \right).$

#### Proof

We can write the assertions illustrated by

23 $$ \begin{aligned}    & \left\| {E_{1} \left( {t,~S_{1} \left( t \right)} \right) - E_{1} \left( {t,~S_{2} \left( t \right)} \right)} \right\| = \left\| {\frac{{{\upvarphi }^{\kappa } \left( {{\uprho }_{1}^{{{\upkappa }}} U + {\uprho }_{2}^{{{\upkappa }}} A} \right)}}{K} - \mu ^{\kappa } \left( {S_{1} \left( t \right) - S_{2} \left( t \right)} \right)} \right\| \le ({\upvarphi }^{\kappa } \left( {{\uprho }_{1}^{{{\upkappa }}}  + {\uprho }_{2}^{{{\upkappa }}} } \right) + \mu ^{\kappa } )\left\| {S_{1}  - S_{2} } \right\|, \\     & \left\| {E_{2} \left( {t,~E_{1} \left( t \right)} \right) - E_{2} \left( {t,~E_{2} \left( t \right)} \right)} \right\| \le \left( {\mu ^{\kappa }  + \delta ^{\kappa } } \right)\left\| {E_{1}  - E_{2} } \right\|, \\     & \left\| {E_{3} \left( {t,~U_{1} \left( t \right)} \right) - E_{3} \left( {t,~U_{2} \left( t \right)} \right)} \right\| \le \left( {\mu ^{\kappa }  + \varepsilon _{1}^{\kappa } } \right)\left\| {U_{1}  - U_{2} } \right\|, \\     & \left\| {E_{4} \left( {t,~A_{1} \left( t \right)} \right) - E_{4} \left( {t,~A_{2} \left( t \right)} \right)} \right\| \le \left( {{\upmu }^{\kappa }  + d^{\kappa }  + \varepsilon _{2}^{\kappa } } \right)\left\| {I_{{C1}}  - I_{{C2}} } \right\|, \\     & \left\| {E_{5} \left( {t,~R_{1} \left( t \right)} \right) - E_{5} \left( {t,~R_{2} \left( t \right)} \right)} \right\| \le {\upmu }^{\kappa } \left\| {R_{1}  - R_{2} } \right\|. \\  \end{aligned} $$

Therefore, we proved that

24 $$ E\left( {t, W_{1} \left( t \right)} \right) - E\left( {t, W_{2} \left( t \right)} \right) \le {\Gamma }W_{1} - W_{2} , $$

where $$\Gamma = \max \left( {{{\upvarphi }}\left( {{\uprho }_{1}^{{\upkappa }} + {\uprho }_{2}^{{\upkappa }} } \right),{ }\left( {{\upmu }^{{\upkappa }} + {\updelta }^{{\upkappa }} } \right),\left( {{\upmu }^{{\upkappa }} + {\upvarepsilon }_{1}^{{\upkappa }} } \right),\left( {{\upmu }^{{\upkappa }} + {\text{d}}^{{\upkappa }} + {\upvarepsilon }_{2}^{{\upkappa }} } \right),{\upmu }^{{\upkappa }} } \right).$$

#### Lemma 3

From Eq. (19), the dynamical system (17) with (18) one can prove that the system has a unique solution $W\left( t \right) \in {\mathcal{F}}_{b}^{0} \left( J \right).$

#### Proof

We can show the uniqueness in Lemma 3 using Picard–Lindelöf criteria with the corresponding fixed point theory. The dynamical system (17) with initial host population illustrated in (18) can be illustrated by $W\left( t \right) = T\left( {W\left( t \right)} \right)$ where $T$ represented the Picard operator given by

$$ T: {\mathcal{F}}_{b}^{0} \left( {J,{\mathbb{R}}^{5} } \right) \to {\mathcal{F}}_{b}^{0} \left( {J,{\mathbb{R}}^{5} } \right),\;T\left[ {W\left( t \right)} \right] = W\left( 0 \right) + \frac{1}{{{\Gamma }\left( \kappa \right)}}\mathop \smallint \limits_{0}^{t} E\left( {s, W\left( s \right)} \right)\left( {t - s} \right)^{\kappa - 1} ds $$

Furthermore we have

$$  \begin{aligned}   \left\| {T\left( {W_{1} \left( t \right)} \right) - T\left( {W_{2} \left( t \right)} \right)} \right\| &  = \left\| {\frac{1}{{{{\Gamma }}\left( \kappa  \right)}}\mathop \smallint \limits_{0}^{t} \left( {t - s} \right)^{{\kappa  - 1}}  \times \left[ {E\left( {s,~W_{1} \left( s \right)} \right) - W\left( {s,~W_{2} \left( s \right)} \right)} \right]} \right\| \\     &  \le \frac{1}{{{{\Gamma }}\left( \kappa  \right)}}\mathop \smallint \limits_{0}^{t} \left( {t - s} \right)^{{\kappa  - 1}} ds \times \left\| {E\left( {s,~W_{1} \left( s \right)} \right) - E\left( {s,~W_{2} \left( s \right)} \right)} \right\| \\     &  \le \frac{{{\Gamma }}}{{{{\Gamma }}\left( \kappa  \right)}}\mathop \smallint \limits_{0}^{t} \left( {t - s} \right)^{{\kappa  - 1}} ds \le \frac{{{\Gamma }}}{{\kappa {{\Gamma }}\left( \kappa  \right)}}T. \\  \end{aligned}   $$

If we have $\frac{{\Gamma }}{{\kappa {\Gamma }\left( \kappa \right)}}T < 1$, then $T$ shows a contraction, therefore, the dynamical system stated in (17) with initial host population (18) has a unique solution.

### The positive invariant region

In this sub-section, we need to prove the positivity and boundedness of the dynamical system (16) solutions to investigate the mathematical and epidemiological well-posedness of the dynamical system.

#### Theorem 1

The region given by $${\text{B}} = \left\{ {\begin{array}{*{20}c} {\left( {S,E,U,A,R} \right) \in {\mathbb{R}}_{ + }^{5} ,K\left( t \right) \le \frac{{{\Delta }^{\kappa } }}{{\mu^{\kappa } }}} \\ \end{array} } \right\}$$ is positively invariant and bounded for each ${\text{t}} \in \left[ {0,{\text{T}}_{0} } \right]$ where ${\text{T}}_{0} > 0.$

#### Proof

Using the dynamical system (16) we have derived the following expressions

$$^{{\text{C}}} D_{t}^{\kappa } S|_{S = 0} { = }\Delta^{\kappa } \ge 0, $$

$$^{{\text{C}}} D_{t}^{\kappa } E|_{E = 0} = \lambda_{T} {\text{S}} \ge 0, $$

$$^{{\text{C}}} D_{t}^{\kappa } U|_{U = 0} = \pi^{\kappa } \delta^{\kappa } E \ge 0, $$

$$^{{\text{C}}} D_{t}^{\kappa } A|_{A = 0} = \left( {1 - \pi^{\kappa } } \right)\delta^{\kappa } E \ge 0, $$

$$^{{\text{C}}} D_{t}^{\kappa } R|_{R = 0} = \varepsilon_{1}^{\kappa } U + \varepsilon_{2}^{\kappa } A \ge 0. $$

Given that $\left( {S\left( 0 \right),E\left( 0 \right),U\left( 0 \right),A\left( 0 \right),R\left( 0 \right)} \right) \in {\mathbb{R}}_{ + }^{5}$ and the parameters of the dynamical system are all positive then by applying Proposition 5 and Lemma 1 the solutions of fractional order model (16) represented by $\left( {S\left( t \right),E\left( t \right),U\left( t \right),A\left( t \right),R\left( t \right)} \right)$ enter in the space ${\mathbb{R}}_{ + }^{5}$. This, one can justify that the region ${\mathbb{R}}_{ + }^{5}$ is positively invariant. Here we add all the equations illustrated in (16) and derive the equation given by $^{{\text{C}}} D_{t}^{\kappa } K\left( t \right) =$ $^{{\text{C}}} D_{t}^{\vartheta \kappa } S$ + $^{{\text{C}}} D_{t}^{\kappa } E$ + $D_{t}^{\kappa } U$ + $^{{\text{C}}} D_{t}^{\kappa } A$ + $^{{\text{C}}} D_{t}^{\kappa } R = \Delta^{\kappa } - \mu^{\kappa } K\left( t \right) - d^{\kappa } A$.

$$ \Rightarrow D_{t}^{\kappa } K\left( t \right) \le \Delta^{\kappa } - \mu^{\kappa } K\left( t \right). $$

Based on the Laplace transformation criteria stated in Proposition 3 and Proposition 4 we computed the result given by ${\mathcal{L}}\left( {D_{t}^{\kappa } K\left( t \right)} \right) \le \frac{{\Delta^{\kappa } }}{S} - \mu^{\kappa } {\mathcal{L}}\left( {K\left( t \right)} \right)$. Let us simplify it and put the result given by ${\mathcal{L}}\left( {K\left( t \right)} \right) \le \frac{{\Delta^{\kappa } {\text{S}}^{ - 1} }}{{S^{\kappa } + d^{\kappa } }} + \frac{{{\text{S}}^{\kappa - 1} K\left( 0 \right)}}{{S^{\kappa } + \mu^{\kappa } }}$. Using Definition 6 and the inverse Laplace transform operation we derived the expression $K\left( t \right) \le K\left( 0 \right)E_{\kappa } \left( { - \mu^{\kappa } t^{\kappa } } \right) + \frac{{\Delta^{\kappa } }}{{\mu^{\kappa } }}\left( {1 - E_{\kappa } \left( { - \mu^{\kappa } t^{\kappa } } \right)} \right)$. Hence, whenever $K\left( 0 \right) \le \frac{{\Delta^{\kappa } }}{{\mu^{\kappa } }}$, then $0 < K\left( t \right) \le \frac{{\Delta^{\kappa } }}{{\mu^{\kappa } }}$ for each time $t \ge 0$ and the total host population denoted by $K\left( t \right)$ is bounded in the region given by $${\text{B}} = \left\{ {\begin{array}{*{20}c} {\left( {S,E,U,A,R} \right) \in {\mathbb{R}}_{ + }^{5} ,K\left( t \right) \le \frac{{\Delta^{\kappa } }}{{\mu^{\kappa } }}} \\ \end{array} } \right\}.$$

### Reproduction number and equilibrium points

The fractional order dynamical system (17) disease-free equilibrium point is calculated by making all the equation zero as $^{{\text{C}}} D_{t}^{\kappa } S\left( t \right) =$ $^{{\text{C}}} D_{t}^{\kappa } E\left( t \right)$ = $^{{\text{C}}} D_{t}^{\kappa } U\left( t \right)$ = $^{{\text{C}}} D_{t}^{\kappa } A\left( t \right)$ = $^{{\text{C}}} D_{t}^{\kappa } R\left( t \right)$ = 0 such that $E = U = A = R = 0.$ Computing the equations and simplifying the result we have determined the required disease-free equilibrium point by $E_{T}^{0} = (S^{0} ,E^{0} ,U^{0} ,A^{0} ,R^{0}$) = $\left( {\frac{{\Delta^{\kappa } }}{{\mu^{\kappa } }}{ },0,0,0,0{ }} \right)$.

The fractional order dynamical system (17) of *Tinea capitis* infection basic reproduction number represented by ${\mathcal{R}}_{0}^{\kappa }$ has crucial epidemiological factors and plays significant role in mathematical epidemiology. Applying the criteria derived by Van den Driesch and Warmouth illustrated in^34^ we have calculated the basic reproduction number of *Tinea capitis* spreading ${\mathcal{R}}_{0}^{\kappa }$ given by

$$ \begin{aligned} & {\mathcal{R}}_{0}^{\kappa } = \frac{1}{{\delta^{\kappa } + \mu^{\kappa } }}\left( {{\mathcal{R}}_{1}^{\kappa } + {\mathcal{R}}_{2}^{\kappa } + {\mathcal{R}}_{3}^{\kappa } } \right)\;{\text{where}}\; {\mathcal{R}}_{1}^{\kappa } = \frac{{\phi^{\kappa } \delta^{\kappa } \rho_{1}^{\kappa } \left( {1 - \pi^{\kappa } } \right)}}{{\varepsilon_{1}^{\kappa } + \mu^{\kappa } }},\;{\mathcal{R}}_{2}^{\kappa } = \frac{{\phi^{\kappa } \delta^{\kappa } \rho_{2}^{\kappa } \pi^{\kappa } }}{{d^{\kappa } + \varepsilon_{2}^{\kappa } + \mu^{\kappa } }}, \\ & {\text{and}}\;{\mathcal{R}}_{3}^{\kappa } = \frac{{\phi^{\kappa } \delta^{\kappa } \rho_{2}^{\kappa } (1 - \pi^{\kappa } )}}{{\left( {\varepsilon_{1}^{\kappa } + \mu^{\kappa } } \right)\left( {d^{\kappa } + \varepsilon_{2}^{\kappa } + \mu^{\kappa } } \right)}}. \\ \end{aligned} $$

Here we have computed and simplifying the result to determine the endemic equilibrium point of the dynamical system (16) by setting its right hand side equal to zero. Let $E_{T}^{*} = \left( {S^{*} ,E^{*} ,U^{*} ,A^{*} ,R^{*} } \right)$ be the endemic equilibrium point of the fractional order dynamical system (16) and solving for each of $S^{*} ,E^{*} ,U^{*} ,A^{*} ,R^{*}$ we have calculated the required unique endemic equilibrium point whenever ${\mathcal{R}}_{0}^{\kappa } > 1$ and is given by

25 $$ \begin{aligned} & E^{*} = \frac{{\Delta^{\kappa } \left( {\mathcal{R}_{0}^{\kappa } - 1} \right)}}{{\left( {\delta^{\kappa } + \mu^{\kappa } } \right)\left( {\mathcal{R}_{0}^{\kappa } - 1} \right) + \left( {\mu^{\kappa } + \frac{{\mu^{\kappa } }}{{\phi^{\kappa } \rho_{1}^{\kappa } }} + \frac{{\varepsilon_{1}^{\kappa } }}{{\mu^{\kappa } {{\upvarphi }}^{\kappa } \rho_{1}^{\kappa } }}} \right)\mathcal{R}_{1}^{\kappa } + \left( {\frac{1}{{\phi^{\kappa } \rho_{2}^{\kappa } }} + \frac{{\varepsilon_{2}^{\kappa } }}{{\mu^{\kappa } \phi^{\kappa } \rho_{2}^{\kappa } }}} \right)(\mathcal{R}_{2}^{\kappa } + \mathcal{R}_{3}^{\kappa } )}}, \\ & S^{*} = \frac{{1 + \left( {\delta^{\kappa } + \mu^{\kappa } } \right)\mathcal{R}_{0}^{\kappa } }}{{{\mathcal{R}}_{0}^{\kappa } - 1}}E^{*} ,U^{*} = \frac{{\mathcal{R}_{1}^{\kappa } }}{{\phi^{\kappa } \rho_{1}^{\kappa } }}E^{*} ,\;A^{*} = \left( {\frac{{\mathcal{R}_{2}^{\kappa } }}{{\phi^{\kappa } \rho_{2}^{\kappa } }} + \frac{{\mathcal{R}_{3}^{\kappa } }}{{\phi^{\kappa } \rho_{2}^{\kappa } }}} \right)E^{*} , \\ & {\text{and}}\;R^{*} = \left( {\frac{{\varepsilon_{1}^{\kappa } }}{{\mu^{\kappa } \phi^{\kappa } \rho_{1}^{\kappa } }}\mathcal{R}_{1}^{\kappa } + \frac{{\varepsilon_{2}^{\kappa } }}{{\mu^{\kappa } \phi^{\kappa } \rho_{2}^{\kappa } }}\left( {\mathcal{R}_{2}^{\kappa } + \mathcal{R}_{3}^{\kappa } } \right)} \right)E^{*} . \\ \end{aligned} $$

### Disease-free equilibrium point local and global stability

#### Theorem 2

The disease-free equilibrium point $E_{T}^{0}$ of for the fractional order dynamical system (16) has local asymptotic stability whenever ${\mathcal{R}}_{0}^{\kappa } < 1$ and unstable whenever ${\mathcal{R}}_{0}^{\kappa } > 1$.

#### Proof

The dynamical system (16) disease-free equilibrium point $$ E_{T}^{0} = (S^{0} ,,E^{0} ,U^{0} ,A^{0} ,R^{0}$$) = $$ \left( {\frac{{{\text{\rm K}}^{\vartheta } }}{{d^{\vartheta } }}{ },0,0,0,0{ }} \right) $$ local stability has been examined by using the method explained in^38^. For simplicity of computations of the stability analysis, for the fractional order dynamical system (16) ignore the last equation involving $${\text{R}}$$ since it does not occurs in the remaining equations. The dynamical system (16) Jacobian matrix at the disease-free equilibrium point can be calculated and is given by

$$ J\left( {E_{T}^{0} } \right) = \left( {\begin{array}{*{20}c} { - {\upmu }^{\kappa } } & 0 & { - {\Delta }^{\kappa } \kappa } & { - \phi^{\kappa } \rho_{2}^{\kappa } } \\ 0 & {{ } - (\delta^{\kappa } + \mu^{\kappa } )} & {{{\upvarphi }}^{\kappa } \rho_{1}^{\kappa } } & {\phi^{\kappa } \rho_{2}^{\kappa } } \\ {{ }0} & {\delta^{\vartheta } \left( {1 - \pi^{\kappa } } \right)} & { - \left( {\varepsilon_{1}^{\kappa } + \mu^{\kappa } } \right)} & 0 \\ 0 & {\delta^{\kappa } \pi^{\kappa } } & 0 & { - \left( {d^{\kappa } + \varepsilon_{2}^{\kappa } + \mu^{\kappa } } \right)} \\ \end{array} } \right). $$

Solving the $\det \left( {J\left( {E_{T}^{0} } \right) - \lambda I_{4} } \right) = 0$ using the Jacobian matrix $J\left( {E_{T}^{0} } \right)$ we have computed the eigenvalues of $J\left( {E_{STC}^{0} } \right)$ determined as $\lambda_{1} = -\upmu ^{\kappa }$, $\lambda_{2} = - \left( {{\upmu }^{\kappa } + \delta^{\kappa } } \right)$, $\lambda_{3} = - \left( {\varepsilon_{1}^{\kappa } + \mu^{\kappa } } \right){\mathcal{R}}_{1}^{\kappa } ,$ and $\lambda_{4} = - \left( {d^{\kappa } + \varepsilon_{2}^{\kappa } + \mu^{\kappa } } \right)\frac{{1 - {\mathcal{R}}_{0}^{\vartheta } }}{{1 - {\mathcal{R}}_{1}^{\kappa } }}$, hence every eigenvalue has negative real part provided ${\mathcal{R}}_{0}^{\kappa } < 1$ and the hence the disease-free equilibrium point $E_{T}^{0}$ is locally asymptotically stable.

### Equilibrium points global stabilities

#### Theorem 3

- Let ${\text{y}}\left( {\text{t}} \right)$ be real valued , continuous and differentiable function then for any for any ${\text{t }} \ge {\text{ T}},$ we do have $\frac{1}{2}{}_{{\text{T}}}^{{\text{C}}} {\text{D}}_{{\text{t}}}^{{\upkappa }} \left( {{\text{y}}^{2} \left( {\text{t}} \right)} \right) \le {\text{y}}\left( {\text{t}} \right){}_{{\text{T}}}^{{\text{C}}} {\text{D}}_{{\text{t}}}^{{\upkappa }} {\text{y}}\left( {\text{t}} \right)$ for all $0 < {\upkappa } \le 1{ }$ ^51^.
- Let ${\text{y}}\left( {\text{t}} \right)$ be real valued, positive, continuous and differentiable function then for any for any ${\text{t }} \ge {\text{ T}},$ we do have ${}_{{\text{T}}}^{{\text{C}}} {\text{D}}_{{\text{t}}}^{{\upkappa }} \left[ {{\text{y}}\left( {\text{t}} \right) - {\text{y}}^{*} - {\text{y}}^{*} \ln \left( {\frac{{{\text{y}}\left( {\text{t}} \right)}}{{{\text{y}}^{*} }}} \right)} \right] \le \left( {1 - \frac{{{\text{y}}\left( {\text{t}} \right)}}{{{\text{y}}^{*} }}} \right){}_{{\text{T}}}^{{\text{C}}} {\text{D}}_{{\text{t}}}^{{\upkappa }} {\text{y}}\left( {\text{t}} \right),{\text{ y}}^{*} \in {\mathbb{R}}_{ + } ,$ ${\text{for all}}$ $0 < {\upkappa } \le 1$ ^51^.

#### Theorem 4

The fractional order dynamical system (17) disease-free equilibrium point illustrated by ${\text{E}}_{{\text{T}}}^{0} = \left( {{\text{S}}^{0} ,{\text{E}}^{0} ,{\text{U}}^{0} ,{\text{A}}^{0} ,{\text{R}}^{0} } \right)$ = ${ }\left( {\frac{{{\Delta }^{{\upkappa }} }}{{{\upmu }^{{\upkappa }} }}{ },0,0,0,0{ }} \right)$ is globally asymptotically stable provided that ${\mathcal{R}}_{0}^{{\upkappa }} < 1$ and unstable provided that ${\mathcal{R}}_{0}^{\vartheta } > 1.$

#### Proof

Let us formulate the representative Lyapunov function defined by

$$ {\mathbb{L}}\left( t \right) = \frac{{{\Gamma }\left( \kappa \right)}}{{2{\text{S}}^{0} }}E^{2} \left( t \right) + \frac{{\phi^{\kappa } \rho_{1}^{\kappa } {\Gamma }\left( \kappa \right)}}{{2{\text{S}}^{0} \left( {\varepsilon_{1}^{\kappa } + \mu^{\kappa } } \right)}}U^{2} \left( t \right) + \frac{{\phi^{\kappa } \rho_{2}^{\kappa } {\Gamma }\left( \kappa \right)}}{{2{\text{S}}^{0} \left( {d^{\kappa } + \varepsilon_{2}^{\kappa } + \mu^{\kappa } } \right)}}A^{2} \left( t \right) $$

where $${\text{S}}^{0} = \frac{{{\Delta }^{{\upkappa }} }}{{\upmu ^{{\upkappa }} }}$$.

The representative Lyapunov function ${\mathbb{L}}\left( t \right)$ is positive definite and continuous for all ${\text{t }} \ge { }0.$

Applying Theorem 4 we do have

$$ \begin{aligned} &^{{\text{C}}} D_{t}^{\kappa } \left( {{ }{\mathbb{L}}\left( t \right)} \right) = \frac{{{\Gamma }\left( \kappa \right)}}{{2{\text{S}}^{0} }}D_{t}^{\kappa } E^{2} ({\text{t}}) + \frac{{\phi^{\kappa } \rho_{1}^{\kappa } {\Gamma }\left( \kappa \right)}}{{2{\text{S}}^{0} \left( {\varepsilon_{1}^{\kappa } + \mu^{\kappa } } \right)}}D_{t}^{\kappa } U^{2} \left( t \right) + \frac{{\phi^{\kappa } \rho_{2}^{\kappa } {\Gamma }\left( \kappa \right)}}{{2{\text{S}}^{0} \left( {d^{\kappa } + \varepsilon_{2}^{\kappa } + \mu^{\kappa } } \right)}}D_{t}^{\kappa } A^{2} \left( t \right), \\ & \quad \le \frac{{{\Gamma }\left( \kappa \right)}}{{{\text{S}}^{0} }}E\left( {\text{t}} \right){ }D_{t}^{\kappa } E({\text{t}}) + \frac{{\phi^{\kappa } \rho_{1}^{\kappa } {\Gamma }\left( \kappa \right)}}{{{\text{S}}^{0} \left( {\varepsilon_{1}^{\kappa } + \mu^{\kappa } } \right)}}U\left( t \right)D_{t}^{\vartheta } A\left( t \right) + \frac{{\phi^{\kappa } \rho_{2}^{\kappa } {\Gamma }\left( \kappa \right)}}{{{\text{S}}^{0} \left( {d^{\kappa } + \varepsilon_{2}^{\kappa } + \mu^{\kappa } } \right)}}A\left( t \right)D_{t}^{\kappa } A\left( t \right). \\ \end{aligned} $$

Since $${\text{B}} = \left\{ {\begin{array}{*{20}c} {\left( {S,E,U,A,R} \right) \in {\mathbb{R}}_{ + }^{5} ,K\left( t \right) \le \frac{{{\Delta }^{\kappa } }}{{\mu^{\kappa } }}} \\ \end{array} } \right\}$$ we do have

$$ \begin{aligned}^{{\text{C}}} D_{t}^{\kappa } \left( {{ }{\mathbb{L}}\left( t \right)} \right) & \le \frac{{{\Delta }^{{\upkappa }} }}{{{\Gamma }\left( \kappa \right){\upmu }^{{\upkappa }} }}\left( {\frac{{{\Gamma }\left( \kappa \right)}}{{{\text{S}}^{0} }}D_{t}^{\kappa } E\left( {\text{t}} \right) + \frac{{\phi^{\kappa } \rho_{1}^{\kappa } {\Gamma }\left( \kappa \right)}}{{{\text{S}}^{0} \left( {\varepsilon_{1}^{\kappa } + \mu^{\kappa } } \right)}}D_{t}^{\kappa } U\left( t \right) + \frac{{\phi^{\kappa } \rho_{2}^{\kappa } {\Gamma }\left( \kappa \right)}}{{{\text{S}}^{0} \left( {d^{\kappa } + \varepsilon_{2}^{\kappa } + \mu^{\kappa } } \right)}}D_{t}^{\kappa } A\left( t \right)} \right), \\ & \le D_{t}^{\kappa } E\left( {\text{t}} \right) + \frac{{\phi^{\kappa } \rho_{1}^{\kappa } }}{{\left( {\varepsilon_{1}^{\kappa } + \mu^{\kappa } } \right)}}D_{t}^{\kappa } U\left( t \right) + \frac{{\phi^{\kappa } \rho_{2}^{\kappa } }}{{\left( {d^{\kappa } + \varepsilon_{2}^{\kappa } + \mu^{\kappa } } \right)}}D_{t}^{\kappa } A\left( t \right)), \\ & \le \left( {\frac{{\phi^{\kappa } \left( {\rho_{1}^{\kappa } U + \rho_{2}^{\kappa } A} \right)S}}{K} - (\delta^{\kappa } + \mu^{\kappa } } \right)E) + \frac{{\phi^{\kappa } \rho_{1}^{\kappa } \left( {\delta^{\kappa } \left( {1 - \pi^{\kappa } } \right)E - \left( {\varepsilon_{1}^{\kappa } + \mu^{\kappa } } \right)U} \right)}}{{\left( {\eta^{\vartheta } + \alpha_{1}^{\vartheta } + d^{\vartheta } } \right)}} \\ & \quad + \;\frac{{\phi^{\kappa } \rho_{2}^{\kappa } \left( {\phi^{\kappa } \pi^{\kappa } E - \left( {d^{\kappa } + \varepsilon_{2}^{\kappa } + \mu^{\kappa } } \right)U} \right)}}{{\left( {\varepsilon_{1}^{\kappa } + \mu^{\kappa } } \right)}} \\ & \le (\phi^{\kappa } \left( {\rho_{1}^{\kappa } U + \rho_{2}^{\kappa } A} \right)\left( {\frac{S}{K} - 1} \right)\frac{{\phi^{\kappa } \rho_{1}^{\kappa } }}{{\left( {\varepsilon_{1}^{\kappa } + \mu^{\kappa } } \right)}}\left( {\delta^{\kappa } \left( {1 - \pi^{\kappa } } \right) + \frac{{\phi^{\kappa } \rho_{2}^{\kappa } \delta^{\kappa } \pi^{\kappa } }}{{\left( {d^{\kappa } + \varepsilon_{2}^{\kappa } + \mu^{\kappa } } \right)}} - (\delta^{\kappa } + \mu^{\kappa } } \right)E \\ & \quad + \;\frac{{{{\upvarphi }}^{\kappa } \rho_{2}^{\kappa } U}}{{\left( {d^{\kappa } + \varepsilon_{2}^{\kappa } + \mu^{\kappa } } \right)}}. \\ \end{aligned} $$

Based on the equilibrium point $U = \frac{{\delta^{\kappa } \left( {1 - \pi^{\kappa } } \right)}}{{\left( {\varepsilon_{1}^{\kappa } + \mu^{\kappa } } \right)}}E$ we determined the result

$$ \begin{aligned}^{{\text{C}}} D_{t}^{\kappa } \left( {{ }{\mathbb{L}}\left( t \right)} \right) & \le (\phi^{\kappa } \left( {\rho_{1}^{\kappa } U + \rho_{2}^{\kappa } A} \right)\left( {\frac{S}{K} - 1} \right) + \frac{{\phi^{\kappa } \rho_{1}^{\kappa } }}{{\left( {\varepsilon_{1}^{\kappa } + \mu^{\kappa } } \right)}}\left( {\delta^{\kappa } \left( {1 - \pi^{\kappa } } \right) + \frac{{\phi^{\kappa } \rho_{2}^{\kappa } \delta^{\kappa } \pi^{\kappa } }}{{\left( {d^{\kappa } + \varepsilon_{2}^{\kappa } + \mu^{\kappa } } \right)}} - (\delta^{\kappa } + \mu^{\kappa } )} \right)E \\ & \quad + \;\frac{{\phi^{\kappa } \rho_{2}^{\kappa } U}}{{\left( {d^{\kappa } + \varepsilon_{2}^{\kappa } + \mu^{\kappa } } \right)}} \le \phi^{\kappa } \left( {\rho_{1}^{\kappa } U + \rho_{2}^{\kappa } A} \right)\left( {\frac{S}{K} - 1} \right) \\ & \quad + \;(\delta^{\kappa } + \mu^{\kappa } )\left( {\frac{{\phi^{\kappa } \rho_{1}^{\kappa } \delta^{\kappa } \left( {1 - \pi^{\kappa } } \right)}}{{(\delta^{\kappa } + \mu^{\kappa } )\left( {\varepsilon_{1}^{\kappa } + \mu^{\kappa } } \right)}} + \frac{{\phi^{\kappa } \rho_{2}^{\kappa } \delta^{\kappa } \pi^{\kappa } }}{{(\delta^{\kappa } + \mu^{\kappa } )\left( {d^{\kappa } + \varepsilon_{2}^{\kappa } + \mu^{\kappa } } \right)}} + \frac{{\phi^{\kappa } \rho_{2}^{\kappa } \delta^{\kappa } (1 - \pi^{\kappa } )}}{{(\delta^{\kappa } + \mu^{\kappa } )\left( {\varepsilon_{1}^{\kappa } + \mu^{\kappa } } \right)\left( {d^{\kappa } + \varepsilon_{2}^{\kappa } + \mu^{\kappa } } \right)}}} \right)E, \\ & \le {{\upvarphi }}^{\kappa } \left( {\rho_{1}^{\kappa } U + \rho_{2}^{\kappa } A} \right)\left( {\frac{S}{K} - 1} \right) + (\delta^{\kappa } + \mu^{\kappa } )\left( {{\mathcal{R}}_{0}^{{\upkappa }} - 1} \right)E. \\ \end{aligned} $$

Because $S \le K$ and ${\mathcal{R}}_{0}^{{\upkappa }} \le 1$ the last explanation (inequality) implies that $^{{\text{C}}} D_{t}^{\kappa } \left( {{ }{\mathbb{L}}\left( t \right)} \right) \le 0$. Moreover, we have $^{{\text{C}}} D_{t}^{\kappa } \left( {{ }{\mathbb{L}}\left( t \right)} \right) = 0$ if and only if $\left( {S,E,U,A,R} \right) = E_{T}^{0} = \left( {S^{0} ,0,0,0,0} \right)$. Thus, the maximum invariant set.

Represented by $$\left\{ {\begin{array}{*{20}c} {\left( {S,E,U,A,R} \right) \in {\mathbb{R}}_{ + }^{5} :D_{t}^{\kappa } \left( {{ }{\mathbb{L}}\left( t \right)} \right) = 0 } \\ \end{array} } \right\}$$ is the singleton set $\left\{ {E_{T}^{0} = \left( {\frac{{{\Delta }^{\kappa } }}{{\mu^{\kappa } }},0,0,0,0} \right)} \right\}$. Therefore, by the LaSalle’s invariance principle the dynamical system (16) disease free-equilibrium point is globally asymptotically stable provided that ${\mathcal{R}}_{0}^{{\upkappa }} < 1.$

#### Theorem 5

The fractional order dynamical system (16) endemic equilibrium point represented by $E_{T}^{*} = ({\text{S}}^{*} ,{\text{E}}^{*} ,{\text{U}}^{*} ,{\text{A}}^{*} ,{\text{R}}^{*} )$ stated in (25) is globally asymptotically stable in the region $${\text{B}} = \left\{ {\begin{array}{*{20}c} {\left( {S,E,U,A,R} \right) \in {\mathbb{R}}_{ + }^{5} ,K\left( t \right) \le \frac{{{\Delta }^{\kappa } }}{{\mu^{\kappa } }}} \\ \end{array} } \right\}$$ provided that ${\mathcal{R}}_{0}^{{\upkappa }} > 1$.

#### Proof

Let $$0 < \kappa \le 1$$ is the order of the dynamical system (16) then we seek to prove that the unique endemic equilibrium point $$E_{T}^{*} $$ is globally asymptotically stable whenever $$\mathcal{R}_{E}^{\kappa } > 1$$. Applying the Lyapunov function development criteria described in references^36,38^, we represent the Lyapunov function defined by

26 $$ \begin{aligned} M\left( t \right) & = \left( {S - S^{*} - S^{*} \ln \left( {\frac{S}{{S^{*} }}} \right)} \right) + \left( {E - E^{*} - E^{*} \ln \left( {\frac{E}{{E^{*} }}} \right)} \right) \\ & \quad + \;\frac{{\phi^{\kappa } \rho_{1}^{\kappa } }}{{\left( {\varepsilon_{1}^{\kappa } + \mu^{\kappa } } \right)}}S^{*} \left( {{\text{U}} - {\text{U}}^{*} - {\text{U}}^{*} \ln \left( {\frac{{\text{U}}}{{{\text{U}}^{*} }}} \right)} \right) + \frac{{\phi^{\kappa } \rho_{2}^{\kappa } }}{{\left( {d^{\varphi } + \varepsilon_{2}^{\kappa } + \mu^{\kappa } } \right)}}S^{*} \left( {{\text{A}} - {\text{A}}^{*} - {\text{A}}^{*} \ln \left( {\frac{{\text{A}}}{{{\text{A}}^{*} }}} \right)} \right). \\ \end{aligned} $$

Using item (b) of Theorem 4 we have the result given by

27 $$ \begin{aligned}^{{\text{C}}} D_{t}^{\kappa } \left( {{ }M\left( t \right)} \right) & \le \left( {1 - \frac{S}{{S^{*} }}} \right)D_{t}^{\kappa } S\left( t \right) + \left( {1 - \frac{E}{{E^{*} }}} \right)D_{t}^{\kappa } E\left( t \right) + \frac{{\phi^{\kappa } \rho_{1}^{\kappa } }}{{\left( {\varepsilon_{1}^{\kappa } + \mu^{\kappa } } \right)}}S^{*} \left( {1 - \frac{{\text{U}}}{{{\text{U}}^{*} }}} \right)D_{t}^{\kappa } {\text{U}}\left( t \right) \\ & \quad + \;\frac{{\phi^{\kappa } \rho_{2}^{\kappa } }}{{\left( {d^{\kappa } + \varepsilon_{2}^{\kappa } + \mu^{\kappa } } \right)}}S^{*} \left( {1 - \frac{{\text{A}}}{{{\text{A}}^{*} }}} \right)D_{t}^{\kappa } {\text{A}}\left( t \right). \\ \end{aligned} $$

Based on the fractional order dynamical system (16) and its endemic equilibrium point computed in (25) we have the results represented by:

28 $$ {\Delta }^{\kappa } = \phi^{\kappa } \left( {\rho_{1}^{\kappa } {\text{U}}^{*} + \rho_{2}^{\kappa } {\text{A}}^{*} } \right)S^{*} + \mu^{\kappa } S^{*} , $$

29 $$ - (\delta^{\kappa } + \mu^{\kappa } ) = \frac{{\phi^{\kappa } \left( {\rho_{1}^{\kappa } {\text{U}}^{*} + \rho_{2}^{\kappa } {\text{A}}^{*} } \right)S^{*} }}{{E^{*} }}, $$

30 $$ \delta^{\kappa } (1 - \pi^{\kappa } ) = \frac{{\left( {\varepsilon_{1}^{\kappa } + \mu^{\kappa } } \right){\text{U}}^{*} }}{{E^{*} }}, $$

31 $$ \delta^{\kappa } \pi^{\kappa } = \frac{{\left( {d^{\kappa } + \varepsilon_{2}^{\kappa } + \mu^{\kappa } } \right){\text{A}}^{*} }}{{E^{*} }}. $$

Substituting expressions from (28) to (31) in (27) and computing it gives the result

$$ \begin{aligned} &^{{\text{C}}} D_{t}^{\kappa } {\text{M}}\left( {\text{t}} \right) \le 2\mu^{\kappa } S^{*} \left( {2 - \frac{S}{{S^{*} }} - \frac{{S^{*} }}{S}} \right) + \phi^{\kappa } \rho_{1}^{\kappa } U^{*} S^{*} \left( {3 - \frac{{S^{*} }}{S} - \frac{{EU^{*} }}{{E^{*} U}} - \frac{{SE^{*} U}}{{S^{*} EU^{*} }}} \right) \\ & \quad + \;\updelta ^{\kappa } \rho_{2}^{\kappa } A^{*} S^{*} \left( {3 - \frac{{S^{*} }}{S} - \frac{{EA^{*} }}{{E^{*} A}} - \frac{{SE^{*} A}}{{S^{*} EA^{*} }}} \right) \\ \end{aligned} $$

then applying the arithmetic–geometric mean conditions we determined the result given by $2 - \frac{S}{{S^{*} }} - \frac{{S^{*} }}{S} \le 0,$ $3 - \frac{{S^{*} }}{S} - \frac{{EU^{*} }}{{E^{*} U}} - \frac{{SE^{*} U}}{{S^{*} EU^{*} }} \le 0$, and $3 - \frac{{S^{*} }}{S} - \frac{{EA^{*} }}{{E^{*} A}} - \frac{{SE^{*} A}}{{S^{*} EA^{*} }} \le 0$. From this result we have determined the result $^{{\text{C}}} D_{t}^{\kappa } {\text{M}}\left( {\text{t}} \right) \le 0.$ And also $^{{\text{C}}} D_{t}^{\kappa } {\text{M}}\left( {\text{t}} \right) = 0$ if and only if $$\begin{array}{*{20}c} {\left( {S,E,U,A,R} \right) = \begin{array}{*{20}c} {\left( {S^{*} ,E^{*} ,U^{*} ,A^{*} ,R^{*} } \right)} \\ \end{array} } \\ \end{array}$$. Hence, the largest positive invariant set in this feasible region which satisfies the condition $\left\{ {\left( {S,E,U,A,R} \right) \in {\mathbb{R}}_{ + }^{5} :D_{t}^{\kappa } {\text{M}}\left( {\text{t}} \right) = 0} \right\}$ is only the singleton set $\left\{ {E^{*} = \left( {S^{*} ,E^{*} ,U^{*} ,A^{*} ,R^{*} } \right)} \right\}$. Therefore, the fractional order dynamical system (16) endemic equilibrium point is globally asymptotically stable provided that ${\mathcal{R}}_{0}^{\kappa } > 1$.

## Formulation of the corresponding optimal control problem

In this sub-section, we consider three time-dependent control measures to extend the dynamical system (16). Suppose ${\text{ r}}_{1} \left( {\text{t}} \right)$,${\text{ r}}_{2} \left( {\text{t}} \right)$ and ${\text{r}}_{3} \left( {\text{t}} \right)$ such that with 0 $\le {\text{r}}_{1} \left( {\text{t}} \right),{\text{r}}_{2} \left( {\text{t}} \right),{\text{r}}_{3} \left( {\text{t}} \right) \le {\text{it}}$ be the measurable Lebesgue controlling functions that represents the control strategies defined by:

1. Prevention measures of *Tinea capitis* infection: The strategy ${\text{r}}_{1} \left( {\text{t}} \right)$ describes the level of *Tinea capitis* prevention efforts in order to minimize the effective contact rate. *Tinea capitisi* preventive measures include washing and do not use other person dressing materials.
2. Treatment measures of *Tinea capitis* infection: The time dependent control strategy denoted by ${\text{r}}_{2} \left( {\text{t}} \right),{\text{r}}_{3} \left( {\text{t}} \right)$ are treatment measures of non-inflammatory and inflammatory infected people respectively.

Depending on the control functions illustrated above the new fractional order optimal control problem of the dynamical system (16) can be re-structured by:

32 $$ \begin{aligned} &^{{\text{C}}} D_{t}^{\kappa } S = {\Delta }^{\kappa } - \left( {\left( {1 - { }r_{1} \left( t \right)} \right){ }\frac{{\phi^{\kappa } \left( {\rho_{1}^{\kappa } U + \rho_{2}^{\kappa } A} \right)}}{K} + \mu^{\kappa } } \right)S, \\ &^{{\text{C}}} D_{t}^{\kappa } E = \left( {1 - { }r_{1} \left( t \right)} \right){ }\frac{{\phi^{\kappa } \left( {\rho_{1}^{\kappa } U + \rho_{2}^{\kappa } A} \right)}}{M} - \left( {\mu^{\kappa } + \delta^{\kappa } } \right)E, \\ &^{{\text{C}}} D_{t}^{\kappa } U = \left( {1 - \pi^{\kappa } } \right)\delta^{\kappa } E - \left( {\mu^{\kappa } + r_{2} \left( t \right)\varepsilon_{1}^{\kappa } } \right)U, \\ &^{{\text{C}}} D_{t}^{\kappa } A = \pi^{\kappa } \delta^{\kappa } E - \left( {\mu^{\kappa } + d^{\kappa } + r_{3} \left( t \right)\varepsilon_{2}^{\kappa } } \right)A, \\ &^{{\text{C}}} D_{t}^{\kappa } R = r_{2} \left( t \right)\varepsilon_{1}^{\kappa } U + r_{3} \left( t \right)\varepsilon_{2}^{\kappa } A - \mu^{\kappa } R, \\ \end{aligned} $$

with initial host population $$ S\left( 0 \right) > 0$$, $$E\left( 0 \right) \ge 0, U\left( 0 \right) \ge 0$$, $$ A\left( 0 \right) \ge 0$$, and $$ R\left( 0 \right) \ge 0, $$ and the limited controlling set given by $${\Delta }_{C} = \left\{ {r_{1} \left( t \right), r_{2} \left( t \right),r_{3} \left( t \right):0 \le r_{1} \left( t \right), r_{2} \left( t \right), r_{3} \left( t \right) \le 1 , t \in \left[ {0,T_{f} } \right]} \right\}$$, where $$T_{f} $$ is the final time of implementing control measures. The objective of the control problem is to minimize the number *Tinea capitis* infected individuals and to maximize the number of recovered individuals under the cost of incorporating control measures. To reduce the number of infected individuals in the community we construct the objective function defined by

33 $$ J\left( {r_{1} , r_{2} ,r_{3} } \right) = \mathop \smallint \limits_{0}^{{T_{f} }} \left( {D_{1} E + D_{2} {\text{U}} + D_{3} {\text{A}} + \frac{{{\Gamma }_{1} }}{2}r_{1}^{2} + \frac{{{\Gamma }_{2} }}{2}r_{2}^{2} + \frac{{{\Gamma }_{3} }}{2}r_{3}^{2} } \right)dt. $$

In order to controlling the number of *Tinea capitis* infected people and the cost to apply prevention and treatment control measures described by $$r_{1} \left( t \right), r_{2} \left( t \right)$$ and $$r_{3} \left( t \right)$$ are minimized subject to the system (33) where the constant $$T_{f}$$ describes the final time, the coefficients $$D_{1} ,D_{2} $$ and $$D_{3} $$ are positive weight constants and $$ \frac{{{\Gamma }_{1} }}{2}, \frac{{{\Gamma }_{2} }}{2}$$ and $$\frac{{{\Gamma }_{3} }}{2}$$ are the measure of relative costs of prevention and treatments associated to the controls $$r_{1} , r_{2} $$ and $$ r_{3}$$, respectively, and also balances the units of the integrand. The aim is to find the optimal values $$r^{*} = \left( {r_{1}^{*} , r_{2}^{*} ,r_{3}^{*} } \right)$$ of the controls $$r = \left( {r_{1} , r_{2} ,r_{3} } \right)$$ such that the corresponding state trajectories $$S^{*} , E^{*} , U^{*} , A^{*} , R^{*} $$ are solution of the Eq. (33) in the given time interval $$\left[ {0,T_{f} } \right]$$ with initial data and minimize the objective functional. In the cost functional, the term $$ D_{1} E $$ refer to the cost related to exposed individuals, the term $$D_{2} {\text{I}}_{A}$$ refer to the cost related to acutely infected individuals and the term $$D_{3} {\text{I}}_{C}$$ refer to the cost related to chronically infected individuals. Also $$D_{i}$$ for $$i = 1,2,3$$ are positive constants that represent the cost of incorporating the three controlling strategies and $${\Gamma }_{i}$$ for $$i = 1,2,3$$ are the corresponding efforts applying to minimize the transmission of the infection and $$T_{f}$$ is the final time of applying the control measures.

The objective of the *Tinea capitis* fractional order optimal control problem constructed in (32) is to investigate the optimal control variable $r\left( t \right)$ that minimize the objective functional given by $\mathop {\min }\limits_{{\overline{r} \in \overline{R}}} J(\overline{r})$, subject to the new optimal control dynamical system stated in (32) with the initial data. The vector $\overline{r} = \{ r_{1} , r_{2} , r_{3} )$ is the controlling vector, and the closed and bounded set

34 $$ \overline{R} = \left\{ {\overline{r} \in \left( {L^{\infty } \left( {\left[ {0,T_{f} } \right]} \right)} \right)^{3} ,\;0 \le r_{i} \le 1,\;i = 1, 2, 3,} \right\} $$

is the set of admissible controls.

### Existence and optimality of the control measures

The fractional order dynamical system (22) with (23) can be re-formulated by

$$^{{\text{C}}} D_{t}^{\kappa } Z = M\left( {t,Y\left( t \right)} \right) + N\left( {t,Z\left( t \right)} \right)\overline{u}, 0 \le t \le T_{f} ,\;Z\left( t \right) = Z_{0} , $$

where $Z\left( t \right) = \left( {S\left( t \right),E\left( t \right),U\left( t \right),A\left( t \right),R\left( t \right)} \right)$ represents the dynamical system state variables, $r\left( t \right) = (r_{1} \left( t \right),r_{2} \left( t \right),r_{3} \left( t \right)$ represents the control functions (variables) in the control problem stated in (32) and

$$ M\left( {t,Z\left( t \right)} \right) = \left[ {\begin{array}{*{20}c} {{\Delta }^{\kappa } - \left( {{ }\frac{{\phi^{\kappa } \left( {\rho_{1}^{\kappa } U + \rho_{2}^{\kappa } A} \right)}}{K} + \mu^{\kappa } } \right)S} \\ {\frac{{\phi^{\kappa } \left( {\rho_{1}^{\kappa } U + \rho_{2}^{\kappa } A} \right)}}{K}S - \left( {\mu^{\kappa } + \delta^{\kappa } } \right)E} \\ {(1 - \pi^{\kappa } )\delta^{\kappa } E - \mu^{\kappa } U} \\ {\pi^{\kappa } \delta^{\kappa } E - \left( {{\upmu }^{\kappa } + d^{\kappa } } \right)A} \\ { - \mu^{\kappa } R} \\ \end{array} } \right],\; N\left( {t,Z\left( t \right)} \right) = \left[ {\begin{array}{*{20}c} {\frac{{\phi^{\kappa } \left( {\rho_{1}^{\kappa } U + \rho_{2}^{\kappa } A} \right)}}{K}{\text{S}}} & 0 & 0 \\ { - \frac{{\phi^{\kappa } \left( {\rho_{1}^{\kappa } U + \rho_{2}^{\kappa } A} \right)}}{K}{\text{S}}} & 0 & 0 \\ 0 & { - \varepsilon_{1}^{\kappa } U} & 0 \\ 0 & 0 & { - \varepsilon_{2}^{\kappa } A} \\ 0 & {\varepsilon_{1}^{\kappa } U} & {\varepsilon_{1}^{\kappa } U} \\ \end{array} } \right]. $$

Here to prove that the existence of the three optimal control strategies we need to prove the conditions illustrated as: The control trajectories are non-empty, the set of admissible controls is convex, bounded and closed, the function defined by $M\left( {t,Z\left( t \right)} \right) + N\left( {t,Z\left( t \right)} \right)$ is bounded in the state varibles and controlling variables, and $D_{1} E + D_{2} {\text{U}} + D_{3} {\text{A}} + \frac{{\Gamma_{1} }}{2}r_{1}^{2} + \frac{{\Gamma_{2} }}{2}r_{2}^{2} + \frac{{\Gamma_{3} }}{2}r_{3}^{2}$ is convex on the admissible control set $\overline{R}$.

*Note* Based on definitions written in the manuscript we have the conditions stated as: For control functions with values $$ r_{1} = 1,$$ $$ r_{2} = 0$$ and $$ r_{3} = 0$$ in the admissible control set $$\overline{R}$$ defined in (34) and the solution $$Z = \left( {S,E,{\text{U}},{\text{A}},R} \right)$$ of the fractional order model (16) with given initial data the set of all the control problem feasible solution is non-empty, based on the definition of the admissible control set $$\overline{R}$$ the control set $$\overline{R}$$ is bounded, closed and convex, based on the existence and uniqueness criteria for the model (16) the model (32) solutions are unique and bounded because $$0 \le r_{i} \le 1,$$ for $$i = 1,2,3.$$

#### Theorem 6

The function defined by $M\left( {t,Z\left( t \right)} \right) + N\left( {t,Z\left( t \right)} \right)\overline{r}$ which satisfies at the solution $\overline{Z} = \left( {S,E,{\text{U}},{\text{A}},R} \right)$ such that

35 $$ M\left( {t,\overline{Z}} \right) + N\left( {t,\overline{Z}} \right) \le max\left( {k_{1} ,k_{2} } \right)\left( {\overline{Z} + \overline{r}} \right), $$

where $$k_{1} = {\text{max}}\left( {1 + \phi^{\kappa } \left( {\rho_{1}^{\kappa } + \rho_{2}^{\kappa } } \right) + \mu^{\kappa } ,\mu^{\kappa } + \delta^{\kappa } ,\mu^{\kappa } + \varepsilon_{1}^{\kappa } ,\mu^{\varepsilon } + d^{\kappa } + \varepsilon_{2}^{\kappa } ,\mu^{\kappa } } \right)$$, and $$k_{2} = {\text{max}}\left( {\phi^{\kappa } \left( {\rho_{1}^{\kappa } + \rho_{2}^{\kappa } } \right), 0,1} \right)$$,

#### Proof

Let us re-write the above matrix $M\left( {t,Z\left( t \right)} \right)$ as

$$ M\left( {t,Z\left( t \right)} \right) = \left[ {\begin{array}{*{20}c} D & 0 & 0 & 0 & 0 \\ {\frac{{\phi^{\kappa } \left( {\rho_{1}^{\kappa } U + \rho_{2}^{\kappa } A} \right)}}{K}} & { - \left( {\mu^{\kappa } + \delta^{\kappa } } \right)} & 0 & 0 & 0 \\ 0 & {(1 - \pi^{\kappa } )\delta^{\kappa } } & {\left( {\mu^{\kappa } + \varepsilon_{1}^{\kappa } } \right)} & 0 & 0 \\ 0 & {\pi^{\kappa } \delta^{\kappa } } & 0 & {\left( {\mu^{\kappa } + d^{\kappa } + \varepsilon_{2}^{\kappa } } \right)} & 0 \\ 0 & 0 & {\varepsilon_{1}^{\kappa } } & {\varepsilon_{2}^{\kappa } } & { - \mu^{\kappa } } \\ \end{array} } \right]\left[ {\begin{array}{*{20}c} S \\ E \\ {\text{U}} \\ {\text{A}} \\ R \\ \end{array} } \right] $$

where $D = \frac{{{\Delta }^{\kappa } }}{S} - \frac{{\phi^{\kappa } \left( {\rho_{1}^{\kappa } U + \rho_{2}^{\kappa } A} \right)}}{M}$. From the matrix $M\left( {t,Z\left( t \right)} \right)$ we have ${\Delta }^{\kappa } \le S$ and since the solution is bounded and we have shown that

$$  \left\| {M\left( {t,\bar{Z}} \right)} \right\| \le {\text{max}}\left( {1 + \varphi ^{\kappa } \left( {\rho _{1}^{\kappa }  + \rho _{2}^{\kappa } } \right) + \mu ^{\kappa } ,\mu ^{\kappa }  + \delta ^{\kappa } ,\mu ^{\kappa }  + \varepsilon _{1}^{\kappa } ,\mu ^{\kappa }  + d^{\kappa }  + \varepsilon _{2}^{\kappa } ,\mu ^{\kappa } } \right)\left\| {\bar{Z}} \right\|.  $$

Using similar process we can show the following

$$  \left\| {M\left( {t,\bar{Z}} \right)} \right\| \le {\text{max}}\left( {\varphi ^{\kappa } \left( {\rho _{1}^{\kappa }  + \rho _{2}^{\kappa } } \right),~~0,1} \right)\left\| {\bar{r}} \right\|.  $$

#### Theorem 7

The function given by ${\mathbb{V}}\left( {t,\overline{Z},\overline{r}} \right) = D_{1} E + D_{2} {\text{I}}_{A} + D_{3} {\text{I}}_{C} + \frac{{\Gamma_{1} }}{2}r_{1}^{2} + \frac{{\Gamma_{2} }}{2}r_{2}^{2} + \frac{{\Gamma_{3} }}{2}r_{3}^{2}$ is convex in the admissible control region $\overline{R}$ and there exists a constant $k$ which is non-negative such that ${\mathbb{V}}\left( {t,\overline{Z},\overline{r}} \right) \ge k\overline{r}$.

#### Proof

For the function ${\mathbb{V}}\left( {t,\overline{Z},\overline{r}} \right)$ we derived the corresponding Hessian matrix given by

$$ {\mathbb{H}} = \left[ {\begin{array}{*{20}c} {2r_{1} } & 0 & 0 \\ 0 & {2r_{2} } & 0 \\ 0 & 0 & {2r_{3} } \\ \end{array} } \right]. $$

Therefore the matrix ${\mathbb{H}}$ is positive definite matrix in the admissible control region $\overline{R}$ and hence ${\mathbb{V}}\left( {t,\overline{Z},r} \right)$ is strictly convex in $r.$ Let $k = {\text{min}}\left( {\frac{{{\Gamma }_{1} }}{2},\frac{{{\Gamma }_{2} }}{2},\frac{{{\Gamma }_{3} }}{2}} \right)$ then ${\mathbb{V}}\left( {t,\overline{Z},\overline{r}} \right) = D_{1} E + D_{2} {\text{I}}_{A} + D_{3} {\text{I}}_{C} + \frac{{{\Gamma }_{1} }}{2}r_{1}^{2} + \frac{{{\Gamma }_{2} }}{2}r_{2}^{2} + \frac{{{\Gamma }_{3} }}{2}r_{3}^{2} \ge \frac{{{\Gamma }_{1} }}{2}r_{1}^{2} + \frac{{{\Gamma }_{2} }}{2}r_{2}^{2} + \frac{{{\Gamma }_{3} }}{2}r_{3}^{2} \ge k\left( {\frac{{{\Gamma }_{1} }}{2}r_{1}^{2} + \frac{{{\Gamma }_{2} }}{2}r_{2}^{2} + \frac{{{\Gamma }_{3} }}{2}r_{3}^{2} } \right)$. Thus, we established the proof.

#### Theorem 8

There is an optimal control point $\overline{r}^{*} = \left( {r_{1}^{*} ,r_{2}^{*} ,r_{3}^{*} } \right)$ and the model associated solutions $\overline{Z}^{*} = \left( {S^{*} ,E^{*} ,U^{*} ,A^{*} ,R^{*} } \right)$ which minimizes the objective function $J\left( {\overline{r}} \right)$ on the admissible control set $\overline{R}$ such that $\mathop {\min }\limits_{{\overline{r} \in \overline{R}}} J(\overline{r}) = J\left( {\overline{r}^{*} } \right)$.

*The optimality necessary condition* The optimality necessary condition required to be fulfilled by the optimal control problem (32) and (33) is adopted from the Pontryagin’s Maximum principle stated in^39^, and it is also fulfilled by changing in to a minimizing Hamiltonian function with respect to the control variables $\left( {r_{1} ,r_{2} ,r_{3} } \right)$. The corresponding Hamiltonian corresponding to (32) and (33) is derived as:

36 $$ \begin{aligned} H\left( {\overline{Z},\overline{r}, {\Delta }^{\kappa } } \right) & = D_{1} E + D_{2} {\text{U}} + D_{3} {\text{A}} + \frac{{\Gamma_{1} }}{2}r_{1}^{2} + \frac{{\Gamma_{2} }}{2}r_{2}^{2} + \frac{{\Gamma_{3} }}{2}r_{3}^{2} \\ & \quad + \;\lambda_{1} \left( {{\Delta }^{\kappa } - \left( {\left( {1 - { }r_{1} \left( t \right)} \right){ }\frac{{\phi^{\kappa } \left( {\rho_{1}^{\kappa } U + \rho_{2}^{\kappa } A} \right)}}{K} + \mu^{\kappa } } \right)S} \right) \\ & \quad + \;\lambda_{2} \left( {\left( {1 - { }r_{1} \left( t \right)} \right){ }\frac{{\phi^{\kappa } \left( {\rho_{1}^{\kappa } U + \rho_{2}^{\kappa } A} \right)}}{K} - \left( {\mu^{\kappa } + \delta^{\kappa } } \right)E} \right) \\ & \quad + \;\lambda_{3} \left( {\left( {1 - \pi^{\kappa } } \right)\delta^{\kappa } E - \left( {\mu^{\kappa } + r_{2} \left( t \right)\alpha_{1}^{\kappa } } \right)U} \right) \\ & \quad + \;\lambda_{4} \left( {\pi^{\kappa } \delta^{\kappa } E - \left( {\mu^{\kappa } + d^{\kappa } + r_{3} \left( t \right)\varepsilon_{2}^{\kappa } } \right)A} \right) + \lambda_{5} \left( {r_{2} \left( t \right)\varepsilon_{1}^{\kappa } U + r_{3} \left( t \right)\varepsilon_{2}^{\kappa } I_{A} - \mu^{\kappa } R} \right), \\ \end{aligned} $$

where $\lambda_{1} \left( t \right), \lambda_{2} \left( t \right), \lambda_{3} \left( t \right), \lambda_{4} \left( t \right),$ and $\lambda_{5} \left( t \right)$ are the co-state variables or adjoint variables.

#### Theorem 9

Let us given the optimal control solutions $r_{i}^{*}$ for $i = 1,2,3$ and the solutions of the optimal control problem (32) that minimizes the objective function (34) in the admissible control region $\overline{R}$, the there are functions $\lambda_{1} , \lambda_{2} ,\lambda_{3} ,\lambda_{4}$ and $\lambda_{5}$ such that

37 $$ \begin{aligned} &^{{\text{C}}} D_{t}^{\kappa } \lambda_{1} = \left( {\lambda_{1} - \lambda_{2} } \right)\left( {1 - r_{1} } \right)\frac{{\phi^{\kappa } \left( {\rho_{1}^{\kappa } U + \rho_{2}^{\kappa } A} \right)}}{K}\left( {1 - \frac{S}{K}} \right) + \mu^{\kappa } \lambda_{1} , \\ &^{{\text{C}}} D_{t}^{\kappa } \lambda_{2} = \left( {\lambda_{2} - \lambda_{1} } \right)\left( {1 - r_{1} } \right)\frac{{\phi^{\kappa } \left( {\rho_{1}^{\kappa } U + \rho_{2}^{\kappa } A} \right)S}}{{K^{2} }} + \lambda_{2} \left( {\mu^{\kappa } + \delta^{\kappa } } \right) - \lambda_{3} \left( {1 - \pi^{\kappa } } \right)\delta^{\kappa } - \lambda_{3} \pi^{\kappa } \delta^{\kappa } - D_{1} , \\ &^{{\text{C}}} D_{t}^{\kappa } \lambda_{3} = \left( {\lambda_{2} - \lambda_{1} } \right)\left( {1 - r_{1} } \right)\left( {\frac{{\phi^{\kappa } \left( {\rho_{1}^{\kappa } U + \rho_{2}^{\kappa } A} \right)S}}{{K^{2} }} - {{\upvarphi }}^{\kappa } \rho_{1}^{\kappa } \frac{S}{K}} \right) + r_{2} \left( {\lambda_{3} - \lambda_{4} } \right) + B\lambda_{3} + \lambda_{4} \varepsilon_{1}^{\kappa } - D_{2} , \\ &^{{\text{C}}} D_{t}^{\kappa } \lambda_{4} = \left( {\lambda_{2} - \lambda_{1} } \right)\left( {1 - r_{1} } \right)\left( {\frac{{\phi^{\kappa } \left( {\rho_{1}^{\kappa } U + \rho_{2}^{\kappa } A} \right)S}}{{K^{2} }} - {{\upvarphi }}^{\kappa } \rho_{2}^{\kappa } \frac{S}{K}} \right) + \left( {\lambda_{4} - \lambda_{5} } \right)\left( {\varepsilon_{2}^{\kappa } + r_{3} } \right) + \lambda_{4} \left( {\mu^{\kappa } + d^{\kappa } } \right) - D_{3} , \\ &^{{\text{C}}} D_{t}^{\kappa } \lambda_{5} = \left( {\lambda_{2} - \lambda_{1} } \right)\left( {1 - r_{1} } \right)\frac{{\phi^{\kappa } \left( {\rho_{1}^{\kappa } U + \rho_{2}^{\kappa } A} \right)S}}{{K^{2} }} + \lambda_{5} \mu^{\kappa } ,\;{\text{where}}\;B = \left( {\mu^{\kappa } + \varepsilon_{1}^{\kappa } } \right). \\ \end{aligned} $$

The transversality conditions of the system (37) are ${\uplambda }_{{\text{i}}}^{*} \left( {{\text{T}}_{{\text{f}}} } \right) = 0$, ${\text{i}} = 1,{ }2,{ } \ldots ,5$, with the Hamiltonian function ${\text{H}}$ defined in Eq. (36). Moreover, the optimal control strategies are determined as:

38 $$ \begin{aligned} r_{1}^{*} \left( t \right) = \min \left\{ {1, {\text{max}}\left[ {0,\frac{{\left( {\lambda_{1} - \lambda_{2} } \right)}}{{\Gamma_{1} }}\frac{{\phi^{\kappa } \left( {\rho_{1}^{\kappa } U + \rho_{2}^{\kappa } A} \right)S}}{M}} \right]} \right\}, \\ & r_{2}^{*} \left( t \right) = \min \left\{ {1, {\text{max}}\left[ {0,\frac{{\left( {\lambda_{3} - \lambda_{5} } \right)}}{{\Gamma_{2} }}U} \right]} \right\}, \\ & r_{3}^{*} \left( t \right) = \min \left\{ {1, {\text{max}}\left[ {0,\frac{{\left( {\lambda_{4} - \lambda_{5} } \right)}}{{\Gamma_{3} }}A} \right]} \right\}, \\ \end{aligned} $$

where $\lambda_{1} \left( t \right), \lambda_{2} \left( t \right), \lambda_{3} \left( t \right), \lambda_{4} \left( t \right), \lambda_{5} \left( t \right)$ and $\lambda_{6} \left( t \right)$ are the co-state variables or adjoint variables and the transversality conditions discussed above.

#### Proof

Let the co-state variables be $\lambda_{1} \left( t \right), \lambda_{2} \left( t \right), \lambda_{3} \left( t \right), \lambda_{4} \left( t \right), \lambda_{5} \left( t \right)$ and the Pontryagin's maximal principle illustrated in reference^39,51^ we can prove the assertion in (38). And also the characterization of each optimal control strategy defined in (38) is computed by solving the following partial differential equations in the interior of the admissible control set $\overline{R}$.

Let $$\overline{r}^{*} = \left( {r_{1}^{*} ,r_{2}^{*} ,r_{3}^{*} } \right)$$ and $$S^{*}$$,$$ E^{*} , $$ $$U^{*}$$,$$ A^{*}$$ and $$R^{*}$$ be the required solustions. Then based on Pontryagin's maximal principle, there exists adjoint-variables that satisfy:

$$- D_{t}^{\kappa } \lambda_{1} = \frac{\partial H}{{\partial r_{1} }}$$, $$\lambda_{1} \left( {t_{f} } \right) = 0$$, $$- D_{t}^{\kappa } \lambda_{2} = \frac{\partial H}{{\partial r_{2} }}$$, $$\lambda_{2} \left( {t_{f} } \right) = 0$$, $$- D_{t}^{\kappa } \lambda_{2} = \frac{\partial H}{{\partial r_{3} }}$$, $$\lambda_{3} \left( {t_{f} } \right) = 0$$. On the interior of the set $$0 < r_{i} < 1 $$ for each $$ i = 1,2,3$$ computed the expressions and we do have the final result

$$ \begin{aligned} & 0 = \frac{\partial H}{{\partial r_{1} }} = M\Gamma_{1} r_{1}^{*} - \left( {\lambda_{1} - \lambda_{2} } \right)\phi^{\kappa } \left( {\rho_{1}^{\kappa } U + \rho_{2}^{\kappa } A} \right)S, \\ & 0 = \frac{\partial H}{{\partial r_{2} }} = \Gamma_{2} r_{2}^{*} - \left( {\lambda_{3} - \lambda_{5} } \right)U, \\ & 0 = \frac{\partial H}{{\partial r_{3} }} = \Gamma_{3} r_{3}^{*} - \left( {\lambda_{4} - \lambda_{5} } \right)A. \\ \end{aligned} $$

Then solving and simplifying these equations we have determined the required results stated in Eq. (3) illustrated by

$$ \begin{aligned} & r_{1}^{*} \left( t \right) = \min \left\{ {1, {\text{max}}\left[ {0,\frac{{\left( {\lambda_{1} - \lambda_{2} } \right)}}{{\Gamma_{1} }}\frac{{\phi^{\kappa } \left( {\rho_{1}^{\kappa } U + \rho_{2}^{\kappa } A} \right)S}}{M}} \right]} \right\}, \\ & r_{2}^{*} \left( t \right) = \min \left\{ {1, {\text{max}}\left[ {0,\frac{{\left( {\lambda_{3} - \lambda_{5} } \right)}}{{\Gamma_{2} }}U} \right]} \right\}, \\ & r_{3}^{*} \left( t \right) = \min \left\{ {1, {\text{max}}\left[ {0,\frac{{\left( {\lambda_{4} - \lambda_{5} } \right)}}{{\Gamma_{3} }}A} \right]} \right\}. \\ \end{aligned} $$

This complete the required prove.

## Sensitivity and numerical analysis

In this sub-section of the study we need to perform the model parameters sensitivity analysis and the numerical simulations such as simulations to investigate the parameter change impacts on the dynamical system, the impact of the fractional order change on the model state variables, and simulations to investigate the impacts of optimal control strategies on the model (32) state variables by applying MATLAB programming codes with Euler forward or/and backward finite difference approach and take the values of the model parameters as: $D_{1} = D_{2} = D_{3} = 13$, $\Gamma_{1} = 36$, $\Gamma_{2} = 39$, $\Gamma_{3} = 41,$ $\pi = 0.51$, $\phi = 0.42$, ${\text{d}} = 0.23$,$\mu = 0.35,\;\Delta = 50$,$\delta = 0.42$,$\varepsilon_{1} = 0.46$,$\varepsilon_{2} = 0.4$, and using different initial host population data.

### Sensitivity analysis

Definition 6: The *Tinea capitis* infection fractional order model basic reproduction number $({\mathcal{R}}_{0}^{\kappa } )$ normalized forward sensitivity index w that depends differentially on a parameter $\omega \user2{ }$ is defined by SEI($\omega$) = $\frac{{\partial {\mathcal{R}}_{0}^{\kappa } }}{\partial \omega }\user2{*}\frac{\omega }{{{\mathcal{R}}_{0}^{\kappa } }}$ ^33,40^.

In this sub-section based on Definition 6 and using the *Tinea capitis* fractional order model (16) parameters values illustrated in “Sensitivity and numerical analysis” section above we have calculated the sensitivity index for the parameters in terms of ${\mathcal{R}}_{0}^{\kappa }$.

Here using the parameter values illustrated in “Sensitivity and numerical analysis” section above, we calculated the *Tinea capitis* fractional order model (16) basic reproduction number as ${\mathcal{R}}_{0}^{\kappa } = 2.76 > 1$ which implies *Tinea capitis* has been spreading in the community. From results illustrated in Table 3 we have observed that the *Tinea capitis* spreading rate $\varphi$ is the most sensitive model parameter which has direct relationship with the basic reproduction number and the recovery rates have also high impact on the basic reproduction number and have an indirect relation with the basic reproduction number.

**Table 3:** Sensitivity indices of $\mathcal{R}_{0}^{\kappa }$.

Sensitivity index	Values
$SEI\left( \varphi \right)$	+ 1
$SEI\left( {\varepsilon_{1} } \right)$	− 0.541
$SEI\left( {\varepsilon_{2} } \right)$	− 0. 694
$SEI\left( {\rho_{1} } \right)$	+ 0.382
$SEI\left( {\rho_{2} } \right)$	+ 0.431
$SEI\left( \delta \right)$	+ 0.512
$SEI\left( d \right)$	+ 0.362
$SEI\left( \pi \right)$	+ 0.421

**Figure 2:**
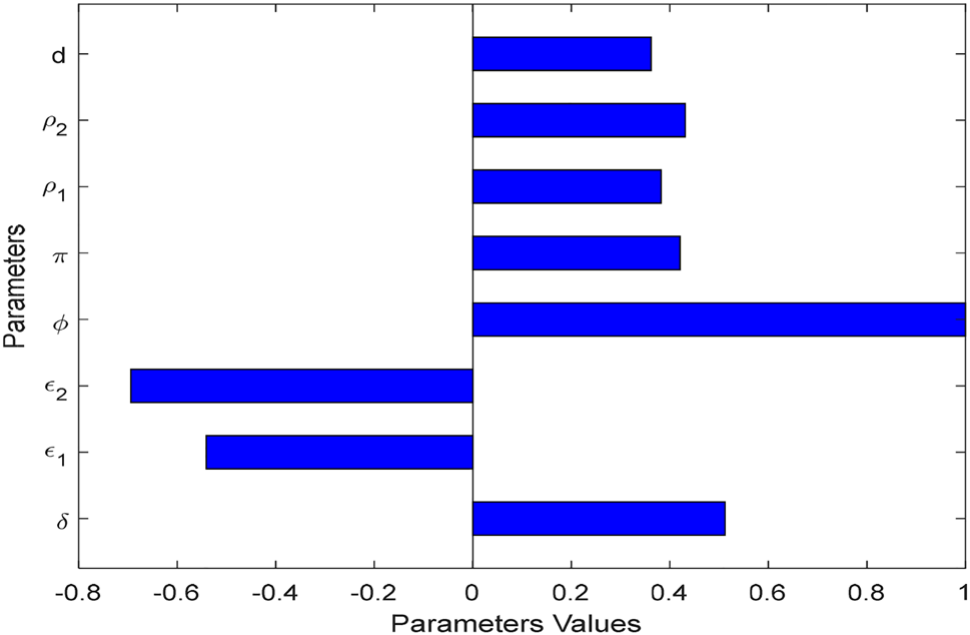
Simulation for sensitivity of the model parameters.

The graph illustrated by Fig. 2 verifies the sensitivity analysis of the dynamical system parameters. From the illustrated figure we observe that the *Tinea capitis* spreading rate $\phi$ is the most sensitive parameter to be controlled in order to tackle the *Tinea capitis* spreading dynamics in the community.

### Numerical simulations

In this sub-section of the study we need to perform the numerical simulations such as simulations to investigate the parameter change impacts on the dynamical system, the impact of the fractional order change on the model state variables, and simulations to investigate the impacts of optimal control strategies on the model (32) state variables by applying MATLAB programming codes with Euler forward or/and backward finite difference approach.

#### Numerical simulations to show the parameters impact

**Figure 3:**
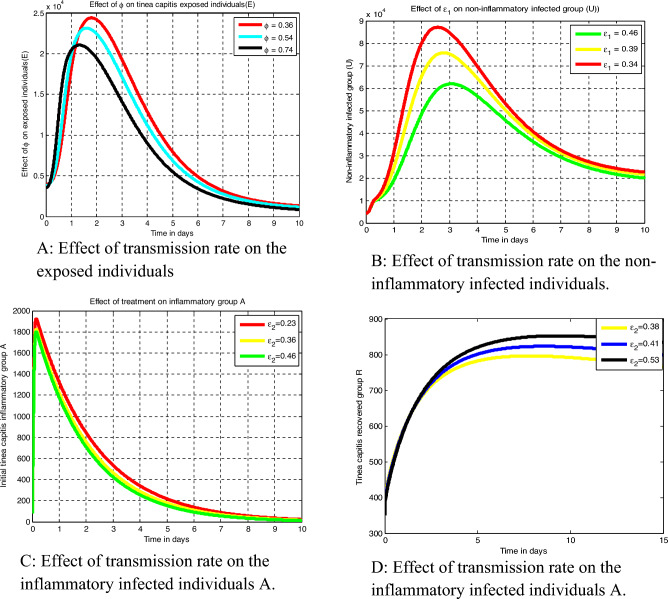
Impacts of parameter changes on the infection status of the model state variables.

The numerical simulation illustrated by Fig. 3A–D reveals that the impacts of parameter changes on the model state variables. From Fig. 3A we observe that whenever the transmission rate increases implies the number of exposed individuals also increases, from Fig. 3B,C we observe that increasing the treatment rates leads to decrease the number of non-inflammatory and inflammatory infected individuals respectively whereas whenever the treatment rate increases implies the *Tinea capitis* recovered group decreases.

#### Numerical simulations to show impact of fractional order changes

**Figure 4:**
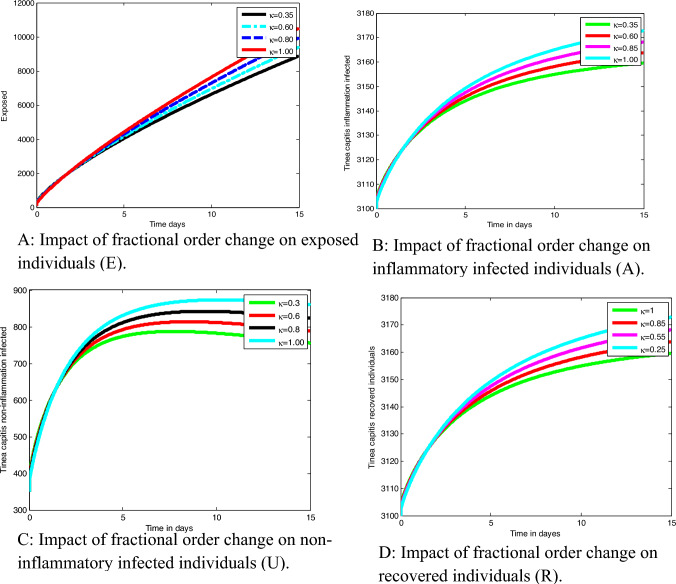
Impacts of fractional order on the infection status of the state variables (memory effects).

The numerical simulation curves illustrated by Fig. 4A–D shown that the effect of fractional order (memory effects) on the infection status of the *Tinea capitis* fractional order model variables. From the result of Fig. 4 one can observe that whenever the fractional order decreases then the number of *Tinea capitis* exposed, non-inflammatory infected, and the inflammatory infected individuals’ decreases due to the memory effect whereas the fractional order decreases implies the number of *Tinea capitis* recovered individuals also increases.

#### Numerical simulations of the optimal control problem

To observe the impact of the controlling strategies and verify the analytical results of the fractional order optimal control problem (32) we carried out the numerical simulation of (32) by applying MATLAB programming codes with Euler forward or/and backward finite difference approach for the following proposed optimal control strategies.

Measure 1: Implementing prevention and non-inflammatory strategies ($r_{1} , r_{2}$) only,

Measure 2: Implementing prevention strategy ($r_{1}$) only,

Measure 3: Implementing prevention and inflammatory infected treatment strategies ($$r_{1} ,$$ $$r_{3 } )$$,

Measure 4: Implementing both treatment strategies ($r_{2} , r_{3}$) simultaneously, and

Measure 5: Implementing all the controlling strategies ($r_{1} , r_{2} , r_{3}$) simultaneously.

##### Effect of Measure 2 ($r_{1} \ne 0$)

**Figure 5:**
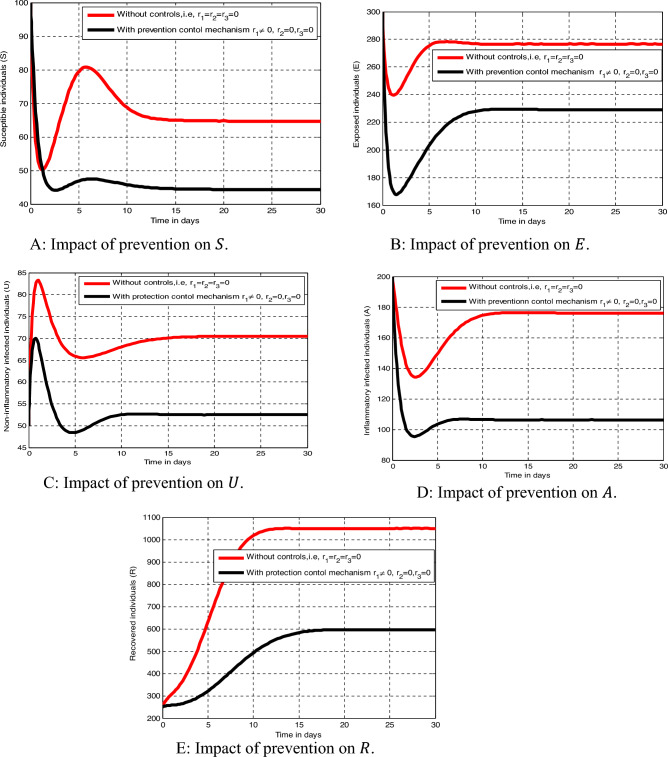
Effect of the control measure $r_{1}$ on the infection status of different population groups at $\kappa = 0.75$.

In this sub-section, we perform numerical simulations without applying prevention or/and treatment control measures in place and by implementing the *Tinea capitis* infection prevention measure (Measure 2) and investigate the impact of prevention strategy i.e., $$r_{1} \ne 0$$, $$ r_{2} = 0$$ and $$r_{3} = 0$$ and making $$\vartheta = 0.75$$. From Fig. 5 we observed the graphical interpretation which shows the impact of the prevention strategy on the *Tinea capitis* transmission dynamics. Whenever we incorporating the control measure $$ r_{1}$$, the exposed individuals illustrated by Fig. 5B, non-inflammatory infected individuals illustrated by Fig. 5C, and inflammatory infected individuals illustrated by Fig. 5D are decreasing significantly, whereas the susceptible individuals illustrated by Fig. 5A and recovered individuals illustrated by Fig. 5E also decreases compared to the case of simulation without controlling strategies.

##### Effects of Measure 1 ($r_{1} \ne 0$ and $r_{2} \ne 0$)

**Figure 6:**
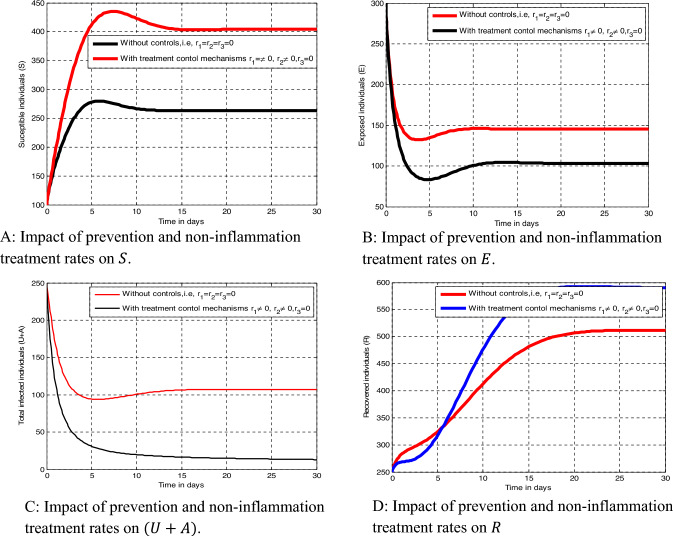
Effect of the control measures $(r_{1} \ne 0 \;{\text{and}}\; r_{2} \ne 0)$ on the infection status of different infected groups at $\kappa = 0.75$.

In this sub-section, we perform numerical simulations without implementing prevention control and non-inflammatory infection treatment control measures $(r_{1} \ne 0$ and $r_{2} \ne 0)$ (Measure 1). From the simulation curve illustrated by Fig. 6 above, Fig. 6A shows decrease of the number of susceptible individuals, Fig. 6B shows individuals in the exposed class are reduced slightly as compared Fig. 5B, the total number of non-inflammatory and inflammatory infected individuals illustrated by Fig. 6C is reduced highly as compared to the first similar classes and the number of recovered individuals illustrated by Fig. 6D increases.

##### Effects of Measure 3 ($r_{1} \ne 0,$ and $r_{3} \ne 0)$

**Figure 7:**
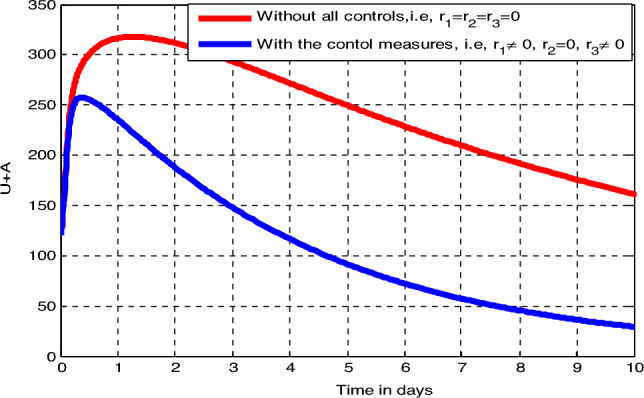
Impact of prevention and inflammatory infected treatment rate on the total number of infected individuals.

Numerical simulation illustrated by Fig. 7 reveals that implementing prevention and *Tinea capitis* non-inflammatory infected individuals leads to a minimization of the total number of *Tinea capitis* infected individuals as compared to the simulation curve without implementing any control measures.

##### Effects of Measure 4 ($r_{2} \ne 0,$ and $r_{3} \ne 0)$

**Figure 8:**
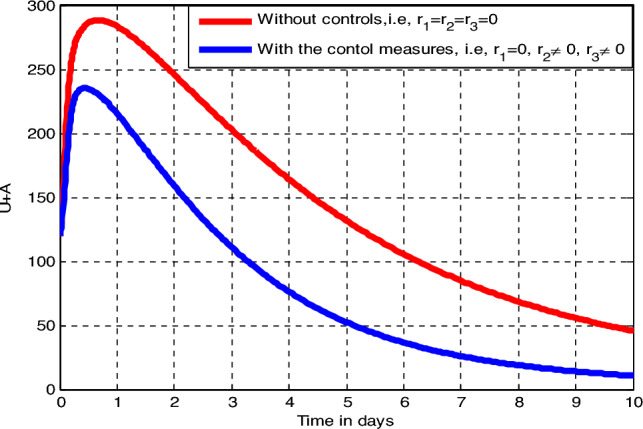
Impact of prevention and inflammatory infected treatment rate on the total number of infected individuals.

Numerical simulation illustrated by Fig. 8 reveals that implementing prevention and *Tinea capitis* inflammatory infected individuals leads to a minimization of the total number of *Tinea capitis* infected individuals as compared to the simulation curve without implementing any control measures.

##### Effects of Measure 5 ($r_{1} , r_{2} \ne 0 ,$ and $r_{3} \ne 0)$

**Figure 9:**
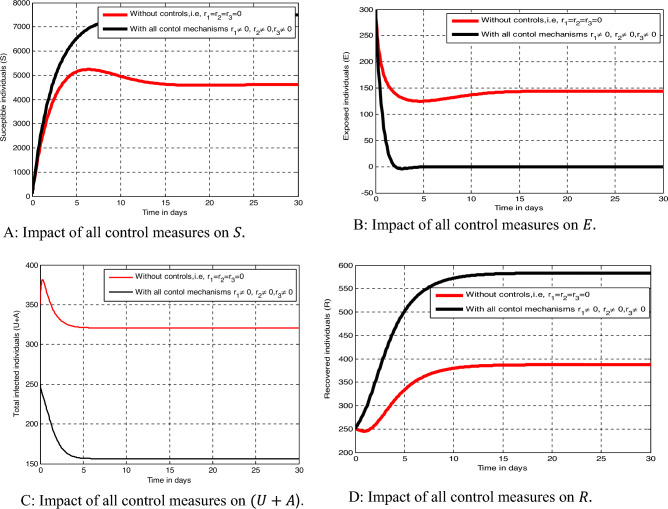
Effect of all the proposed control measures $(r_{1} \ne 0 , r_{2} \ne 0\;{\text{and}}\;r_{3} \ne 0)$ (Measure 5) simultaneously on the infection status of different population groups with $\vartheta = 0.75$.

In this sub-section, we perform numerical simulations without applying all controlling strategies in place and by applying all the possible controlling strategies $(r_{1} \ne 0 , r_{2} \ne 0$ and $r_{3} \ne 0)$ (Measure 5) simultaneously. Here one can compare the effects of different controlling strategies on the infection status of the model state variables. Figure 9A shows the effect of all the proposed controlling strategies on the number of susceptible individuals and has a great impact on increasing the number of susceptible individuals as compared to the number of susceptible individuals in the other strategies. Figure 9B shows the effect of all the proposed controlling strategies on the number of exposed individuals and has a great impact on decreasing the number of exposed individuals as compared to the number of exposed individuals in similar other strategies. Figure 9C shows the effect of all the proposed controlling strategies on the number of infected individuals and has a great impact on decreasing the number of infected individuals as compared to the number of infected individuals in other similar strategies. Figure 9D shows the effect of all the proposed controlling strategies on the number of recovered individuals and has a great impact on increasing the number of recovered individuals as compared to the number of recovered individuals in other similar strategies. Finally, from Fig. 9 we observed the result that implementing all the possible controlling strategies $(r_{1} \ne 0 , r_{2} \ne 0$ and $r_{3} \ne 0)$ (Measure 5) simultaneously makes the number of *Tinea capitis* infected individuals in the community highly decreases after 30 days. And as compared to other strategies this one is the most effective strategy to tackle the spreading rate of *Tinea capitis* infection throughout the community.

## Cost-effective analysis

In this sub-section, we need to carry out the optimal control measures described in “Numerical simulations” section cost-effectiveness analysis to investigate and compare benefits in terms of cost for the control measures applied. To compute the implemented control measures cost-effective analysis, we apply the criteria used by^52^ i.e., the method ICER known as incremental cost-effectiveness ratio which is mathematically defined by ICER = Costs difference in strategies x and y divided by infected averted differences in measures x and y where the numerator incorporates cost differences averted or the cases prevented, interventions costs, and productivity lose costs among others and the denominator is the health outcomes difference of the total infections averted. Based on the results we arrange the effectiveness with increasing order in terms of infection averted quantity and hence the total number of infection averted in measure one, three, two, four and five in an ascending order illustrated in Table 4.

**Table 4:** Aggregate infection averted, aggregate cost, and ICER.

Measures	Aggregate infection averted	Aggregate cost	ICER
1	55,230	25,220	0.4566
3	260,185	33,603	0.0409
2	280,362	33,605	0.0001
4	370,258	40,200	0.0734
5	450,254	60,300	0.2514

Now computing the incremental cost-effectiveness ratios for each possible control measures as: ICER(1) = 25,220/55,230 = 0.4566, ICER(5) = (60,300–40,200)/(450,254–370,258) = 20,100/79,996 = 0.2514, ICER(4) = (40,200–33,604)/(370,258–280,362) = 6596/89,896 = 0.0734,ICER(2) = (33,605–33,603)/(280,362–260,185) = 2/20,177 = 0.0001 and ICER(3) = (33,603–25,220)/(260,185–55,230) = 2/20,177 = 0.0409.

From the result illustrated in Table 5, one can compare control measures 5 and 1 reveal a cost saving of 0.2514 for measure 5 over measure 1. The lower ICER for measure 5 shows that measure 1 is strongly dominated. Which means measure 1 is more costly and less effective than measure 5 hence we should exclude measure 1 from other list.

**Table 5:** Aggregate infection averted, aggregate cost, and ICER.

Measures	Aggregate infection averted	Aggregate cost	ICER
3	260,185	33,603	0.0409
2	280,362	33,605	0.0001
4	370,258	40,200	0.0734
5	450,254	60,300	0.2514

Using the ICER results illustrated in Table 5 we compare values and observe that measure 2 has least value and implementing the control measure 2 or strategy 2 is most cost effective measure we recommend to stakeholder to apply to tackle the *Tinea capitis* spreading dynamics in the community.

## Discussion and conclusion

In this study we have formulated and analyzed the fractional order dynamical system on the fungal *Tinea capitis* infection with time-dependent optimal control measures (strategies). In the qualitative analyses part of the study we have determined all the equilibrium points and the dynamical system basic reproduction number and proved the stabilities of the equilibrium points. In this process, we have re-formulated the corresponding fractional order optimal control problem of *Tinea capitis* infection dynamics in order to minimize the implemented control measure cost while the total number of *Tinea capitis* infected people also needs to be minimized. For the fractional order optimal control problem we have investigated the existence and uniqueness of the optimal controls, and in addition by applying the Pontryagin’s maximum principle we have determined the conditions necessary to investigate the optimal values of the proposed control measures that minimize the transmission of *Tinea capitis* infection and the possible cost of the implemented control measures. Next, we have carried out the sensitivity and numerical analysis of the fractional order model with optimal control measures to investigate the most sensitive model parameters, to show the impact of fractional order on the memory effect, and to verify the qualitative analysis results. The results of these analyses are fundamental to understand how to minimize or eliminate the *Tinea capitis* infection spreading in the community at the cost effective mechanism. From the results of the fractional order optimal control problem numerical simulation part we can suggest that the *Tinea capitis* infection may be eliminated from the community by continuous application of the control measures in a medium time interval. Finally, from the results of cost-effective analysis part the implementing the *Tinea capitis* prevention measure is observed as the most cost-effective strategy. However, implementing other proposed control measures can minimize the number of *Tinea capitis* infected individuals in the community.

For future work, since this study is not exhaustive other potential scholars in the area can modify the proposed model by incorporating additional aspects such as the stochastic approach, age structure of individuals, roles of the community, and fitting the model with appropriate real data.

## Data Availability

Data used to support the findings of this study are included in the article.
